# Mass Spectrometry Reveals Molecular Effects of Citrulline Supplementation during Bone Fracture Healing in a Rat Model

**DOI:** 10.1021/jasms.4c00028

**Published:** 2024-04-29

**Authors:** Sylvia Nauta, Johannes Greven, Martijn Hofman, Ronny Mohren, Dennis M. Meesters, Diana Möckel, Twan Lammers, Frank Hildebrand, Tiffany Porta Siegel, Eva Cuypers, Ron M.A. Heeren, Martijn Poeze

**Affiliations:** † Division of Imaging Mass Spectrometry, Maastricht MultiModal Molecular Imaging (M4i) Institute, Maastricht University, 6229ER Maastricht, The Netherlands; ‡ Division of Traumasurgery, Department of Surgery, Maastricht University Medical Center, 6229HX Maastricht, The Netherlands; § Department of Orthopedics, Trauma and Reconstructive Surgery, University Hospital RWTH Aachen, 52074 Aachen, Germany; ∥ Center of Musculoskeletal Surgery, Bonifatius Hospital Lingen, 49808 Lingen, Germany; ⊥ Department of Genetics & Cell Biology, Maastricht University, 6229ER Maastricht, The Netherlands; # NUTRIM, School for Nutrition and Translational Research in Metabolism, Maastricht University, 6229ER Maastricht, The Netherlands; ▽ Department of Nanomedicine and Theranostics, Institute for Experimental Molecular Imaging, RWTH Aachen University Clinic, 52074 Aachen, Germany

## Abstract

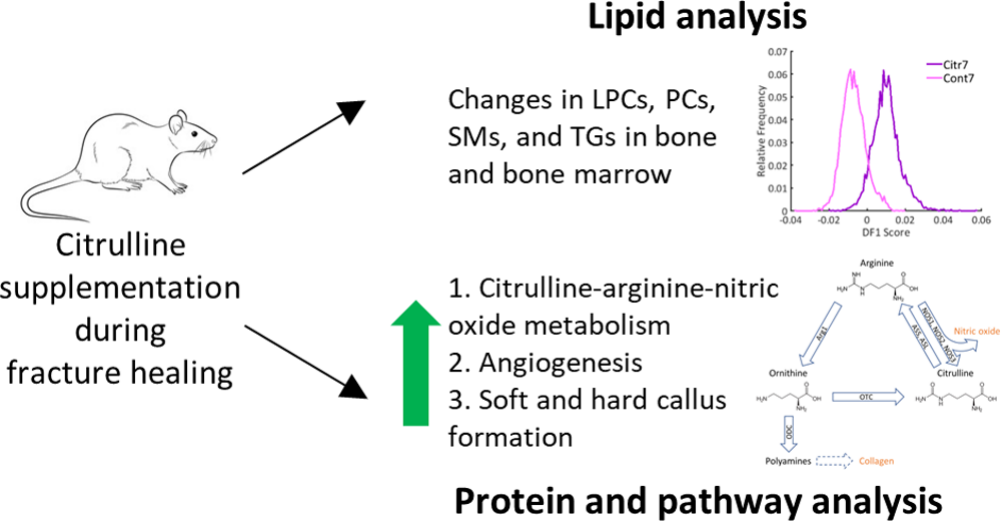

Bone fracture healing is a complex process in which specific molecular knowledge is still lacking. The citrulline–arginine–nitric oxide metabolism is one of the involved pathways, and its enrichment via citrulline supplementation can enhance fracture healing. This study investigated the molecular effects of citrulline supplementation during the different fracture healing phases in a rat model. Microcomputed tomography (μCT) was applied for the analysis of the fracture callus formation. Matrix-assisted laser desorption/ionization mass spectrometry imaging (MALDI-MSI) and liquid-chromatography tandem mass spectrometry (LC-MS/MS) were used for lipid and protein analyses, respectively. μCT analysis showed no significant differences in the fracture callus volume and volume fraction between the citrulline supplementation and control group. The observed lipid profiles for the citrulline supplementation and control group were distinct for the different fracture healing stages. The main contributing lipid classes were phosphatidylcholines (PCs) and lysophosphatidylcholines (LPCs). The changing effect of citrulline supplementation throughout fracture healing was indicated by changes in the differentially expressed proteins between the groups. Pathway analysis showed an enhancement of fracture healing in the citrulline supplementation group in comparison to the control group via improved angiogenesis and earlier formation of the soft and hard callus. This study showed the molecular effects on lipids, proteins, and pathways associated with citrulline supplementation during bone fracture healing, even though no effect was visible with μCT.

## 1 Introduction

Bone fracture healing is a complex but well-defined process.^1−4^ The optimal outcome is the achievement of biological/metabolic and structural restoration of the bone.^1,2,5^ Fracture healing is usually divided into four overlapping phases: (1) hematoma (fibrin clot) formation and inflammation phase, (2) soft callus formation, (3) hard callus formation, and (4) bone remodeling.^2,6,7^ The different phases, the overlap between them, and the crosstalk between different cell types result in a constantly changing cellular and molecular environment around the fracture site.^2,4−6^ The majority of bone fractures will heal normally, but the healing process is impaired in 1–10% of the fractures.^1,5−9^ The treatment of impaired healing cases is a complex, individualized, and lengthy process, despite the improvements in treatment over the past decades.^3,8^ Prevention and treatment of impaired healing can be improved by further insights into fracture healing processes.

Some key molecules that affect the outcome of fracture healing are known and can be associated with impaired fracture healing in case of alternation of the local or systemic regulation.^1−4,10^ The most abundant class of molecules in bone are proteins.^7,11,12^ Different protein classes play an important role during fracture healing, such as pro-inflammatory cytokines, growth and differentiation factors, inhibitory molecules, and angiogenic factors.^1−6^ Lipids are other key molecular players, as they are structural cell components and involved in cellular signaling, inflammation regulation, metabolism, and bone mineralization.^10,13,14^ Lipids are mainly present in bone marrow, but small amounts are present in the mineralized bone as well.^13^

**Figure 1:**
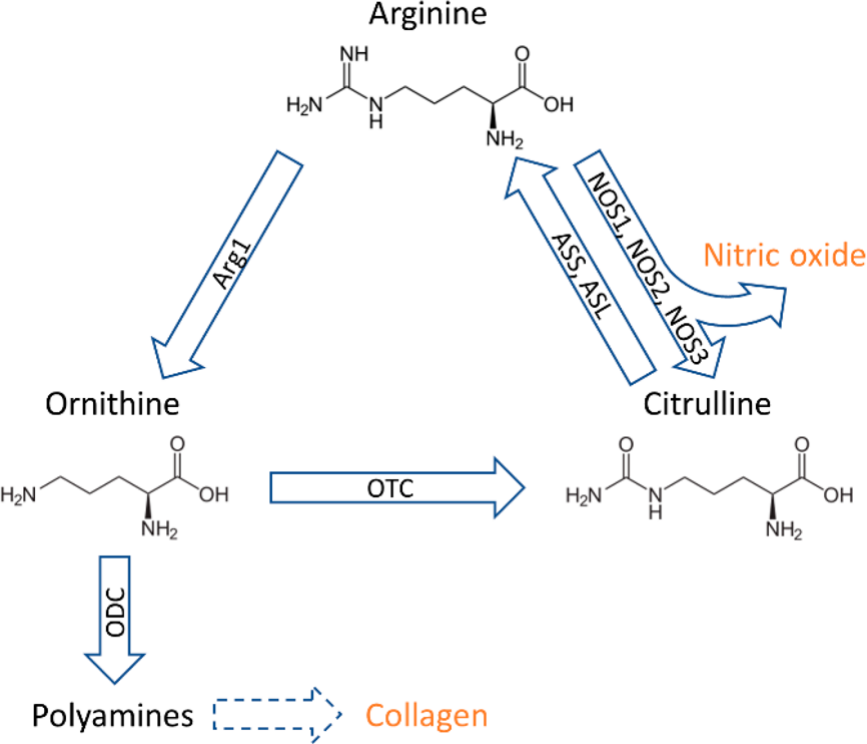
Schematic overview of citrulline–arginine–nitric oxide metabolism. The important molecules in fracture healing are marked orange. Abbreviations: Arg1 = arginase 1; ASS = argininosuccinate synthetase; ASL = argininosuccinate lyase; NOS = nitric oxide synthase; ODC = ornithine decarboxylase; OTC = ornithine transcarbamylase.

The citrulline–arginine–nitric oxide metabolism (see Figure 1) is one of the pathways that is important during fracture healing.^7,15,16^ It has been shown that disruption of this metabolism can result in impaired healing.^8,15−17^ Citrulline is the precursor of arginine.^7,15,16^ Arginine can be converted back into citrulline via the different nitric oxide synthases (NOS1, NOS2, and NOS3), which results in the production of nitric oxide.^7,8,15−21^ Arginine is the only amino acid precursor of nitric oxide.^7,8,16,18^ Nitric oxide is important for fracture healing, as it affects the inflammatory responses, stimulates regulation of bone remodeling, and angiogenesis.^7,8,15−18,20^ In addition, arginine can also be converted into ornithine.^7,8,15,16^ Ornithine is important in collagen synthesis via polyamine production.^7,8,15,16^ Previous studies showed that the supplementation of citrulline can improve fracture healing via stimulation of callus formation and improvement of the inflammatory response and results in improved biomechanical properties.^16,22^ However, the affected and involved molecular pathways are still not fully explored.

Mass spectrometry (MS) is a powerful analytical technique to explore the molecular pathways that are affected by citrulline supplementation. MS allows for the untargeted detection of different molecular classes, including lipids and proteins, from a wide range of tissue types without prior knowledge about the sample composition when conducted.^23−27^ Matrix-assisted laser desorption/ionization mass spectrometry imaging (MALDI-MSI) is often used to study the molecular distributions in tissue sections.^23−25,27,28^ MALDI-MSI has been applied only recently on undecalcified bone tissue, including for lipid analysis, due to the complicated sample preparation protocol.^23−29^ Additionally, tandem mass spectrometry in combination with liquid chromatography (LC-MS/MS) has been shown to be a powerful technique to investigate proteome changes.^30−33^ LC-MS/MS has been applied for the analysis of bone tissue to study proteins, lipids, and drugs in various application fields.^30−35^ Therefore, the combination of lipid analysis with MALDI-MSI and protein analysis with LC-MS/MS can contribute to improving molecular understanding of the effect of citrulline supplementation on bone fracture healing.

The objective of this paper is to study the molecular effects of citrulline supplementation during bone fracture healing on lipid and protein profiles. Fracture healing in the citrulline supplementation group was expected to be enhanced in comparison to the control group. Microcomputed tomography (μCT) was used to study the fracture callus formation. MALDI-MSI was applied for the separate analysis of the lipid profiles in bone and bone marrow. In addition, protein analysis was performed on undecalcified bone tissue using LC-MS/MS and pathway analysis was conducted based on this. Comparisons were performed for these measurements between the citrulline supplementation and control group for the different time points.

## 2 Materials and Methods

### 2.1 Chemicals and Materials

Gelatin, carboxymethyl cellulose (CMC) sodium salt, tragacanth, red phosphorus, α-cyano-4-hydroxycinnamic acid ≥98% (CHCA), sulfosalicylic acid (SSA), ammonium bicarbonate (ABC), dithiothreitol (DTT), iodoacetamide (IAM), and trifluoroacetic acid (TFA, ULC grade) were purchased from Sigma-Aldrich (Zwijndrecht, The Netherlands). Phosphate-buffered saline (PBS) was purchased from Thermo Fisher Scientific Inc. (Waltham, MA). SuperFrost Plus microscopic glass slides were purchased from VWR International (Amsterdam, The Netherlands). Urea was purchased from GE Healthcare (Chicago, IL). The enzyme mixture of trypsin and LysC (mass spectrometry grade) was purchased from Promega (Leiden, The Netherlands). Methanol, acetonitrile (ACN), formic acid (FA), and water with ULC/MS–CC/SFC grade were purchased from Biosolve BV (Valkenswaard, The Netherlands).

### 2.2 Animal Study and Sample Collection

Female, adult Sprague–Dawley rats (approximately 250 g) were obtained from Envigo (Horst, The Netherlands). Female rats were used to keep the load on the bones constant, as they gained less weight than male rats. The animals were at the same menstrual cycle status (determined by vaginal swab) to take into account the protective effect of female hormones in the context of inflammatory reactions. The animals were housed under controlled environmental conditions with a 12-h light–dark cycle and food and water ad libitum. Prior to study inclusion, all the animals were kept in groups for 1 week to allow acclimatization. An intramedullary wire was inserted, and a standardized femoral fracture was generated at the right side under general anesthesia. General anesthesia was induced with ketamine (100 mg/kg ip), xylazine (2%; 10 mg/kg ip) and, if necessary, extended with isoflurane inhalation (2.0–2.5 vol %). The intramedullary wire (1 mm stainless-steel intramedullary Kirschner wire (K-wire), Königsee Implantate GmbH, Allendorf, Germany) was inserted in a retrograde manner. The standardized fractures were created using the blunt guillotine method, as described by Bonnarens and Einhorn.^36^ Fluoroscopic evaluation was performed after placement of the intramedullary pin and the fracture induction. Pre- and postoperative analgesia were ensured with buprenorphine hydrochloride (0.03–0.05 mg/kg sc) 30 min before the operation and every 6 h for the first 24–48 h after the operation, respectively. Afterward, buprenorphine hydrochloride was administered in the same way twice daily during the first 3 weeks. In addition, the drinking water was supplemented with metamizole (1 mL/300 mL) during the first postoperative week. The rats were assigned to the citrulline supplementation (Citr) or control (Cont) group at random. The Citr group received a citrulline-supplemented diet for 14 days postoperative (DPO) and an isocaloric normal diet for the remainder of the study protocol. The citrulline-supplemented diet consisted of 10 g/kg/day citrulline-suspension in sterile water administered per os over a bent metal feeding tube. The Cont group received an isocaloric normal diet throughout the study protocol, as in the previously performed amino acid supplementation study.^16^

**Figure 2:**
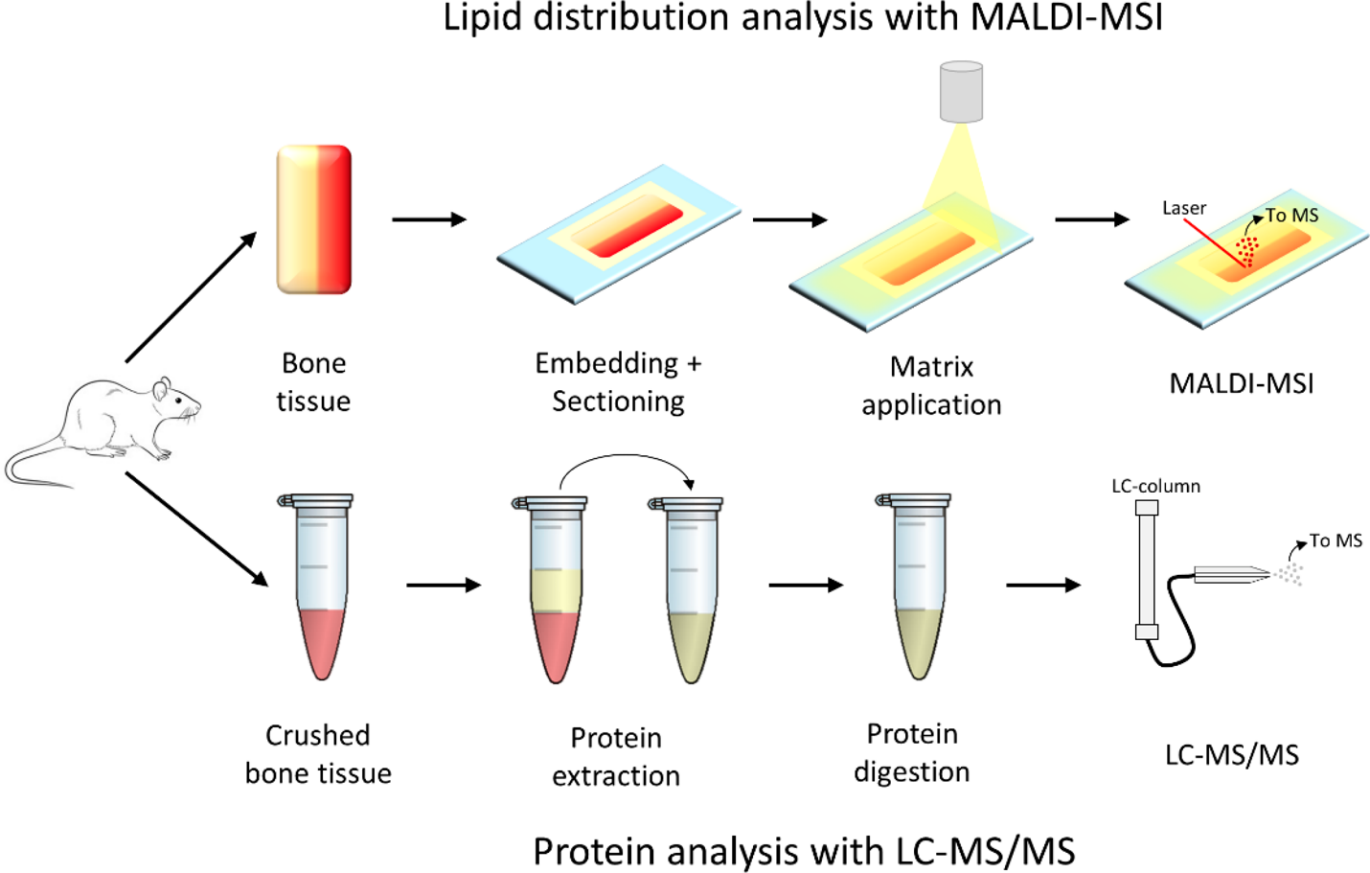
Overview of the analytical workflow combining the analysis of lipid distributions using MALDI-MSI and proteins using LC-MS/MS. Small pieces of bone were used for lipid distribution analysis. These pieces were embedded and sectioned during sample preparation. Matrix was sprayed onto the section before MALDI-MSI analysis. Crushed bone tissue was used for the protein analysis. Proteins were extracted from the crushed bone tissue and subsequently digested using enzymes. The resulting peptides were analyzed with LC-MS/MS.

Samples were collected at four different time points, namely 3, 7, 14, and 28 DPO, corresponding to the different phases in bone fracture healing. In addition, samples were collected for biomechanical testing at 42 DPO, as normal fracture healing in rats is completed at this time point. The power analysis indicated a minimum of four rats per group to achieve a 95% power, and a sample size of six animals per group was selected to compensate for any potential loss of animals. At each time point, six rats per treatment group were exsanguinated by cardiac puncture under anesthesia and analgesia. Three rats (two from Cont14 and one from Citr28) were excluded because of premature death (Supporting Information Table S1). The integral whole femur was collected, soft tissue was removed, and the femurs were washed three times with PBS. The K-wire was carefully removed, and 0.5 cm of the callus at the fracture site was collected. The sample was divided into two pieces with a wire saw while cooling with ice-cold PBS. One piece was used for lipid analysis, and the other piece was homogenized and used for protein analysis. The samples were rapidly frozen and stored at −80 °C until further use. The uncrushed samples were used for lipid analysis with MALDI-MSI, and the homogenized samples were used for protein analysis with LC-MS/MS, as shown in the overview of the analytical workflow in Figure 2. The Ethical Committee of the Governmental Animal Care and Use Committee of the state Nordrhein-Westfalen approved this study (approval number: LANUV NRW 84-02.04.2015.A078).

### 2.3 Microcomputed Tomography

In vivo μCT imaging was obtained at 3, 7, 14, and 28 DPO from the euthanized rats in both the Citr and Cont group. The μCT protocol was optimized to reduce artifacts created by the presence of the K-wire. The K-wire was not removed from the bone before the μCT imaging to prevent any damage to the fragile callus. μCT was performed using a dual-energy gantry-based flat-panel microcomputed tomography scanner (TomoScope 30s Duo, CT Imaging, Erlangen, Germany). The dual-energy X-ray tubes of the μCT were operated at voltages of 40 and 65 kV with currents of 1.0 and 0.5 mA, respectively. Three subscans were performed to cover the entire leg of the rats, and each of the scans acquired 720 projections with 1032 × 1012 pixels during one full rotation with durations of 90 s. Volumetric data sets were reconstructed after acquisition using a modified Feldkamp algorithm with a smooth kernel at an isotropic voxel size of 35 μm. The bone, K-wire, and fracture callus regions were segmented using an automated segmentation method with interactive correction of segmentation errors (Software Imalytics Preclinical^37^). Quantitative analyses were performed for the fracture callus volume, callus volume fraction, bone volume, and bone volume fraction at the fracture side. μCT measurements were performed for groups Citr7 and Cont7 in a nonfrozen state, while the other groups were measured frozen. Data are presented as mean ± standard deviation (STD). The two-tailed unpaired Student’s *t* test was applied for the assessment of statistical significance. *P*-values <0.05 were considered as statistically significant.

### 2.4 Biomechanical Testing

Biomechanical testing was performed at 42 DPO for 6 animals in the Citr and Cont group to analyze the strength of the callus and/or newly formed bone. The fractured femur (right side) as well as the unfractured femur (left control side) were tested. Both ends of the removed femora were embedded in a two-component resin based on methyl methacrylate consisting of a powder (Technovit 3040) and a liquid (Technovit Universal Liquid) component. The embedded femora were tested in a Retroline biomechanical testing device (Zwick Roell AG, Germany). The biomechanical traction test was performed with a traction rate of 1 mm/s = 0.1 N/s and a measurement interval of 0.1 s. The specimens were preloaded with 5 N before imposing the traction. Digital setup and control were performed using TestXpert II software (Zwick Roell AG, Germany), which enabled real-time measurement of the traction force. The obtained parameters were used for the calculation of average load to failure in Newton. Data presentation and statistical testing are the same as for the μCT data.

### 2.5 Lipid Distribution Analysis with MALDI-MSI

#### 2.5.1 Sample Preparation and Matrix Application

The lipid distributions in bone and bone marrow were analyzed with MALDI-MSI. The embedding and sectioning of the samples followed the protocol described by Vandenbosch et al.^27^ In short, pieces of bone with bone marrow (without the K-wire) were embedded in 20% gelatin and 7.5% CMC dissolved in water (w/v) and directly frozen. Tissue sectioning was performed in a Leica CM1860 UV (Wetzlar, Germany) using a Shandon tungsten carbide D-profile knife (Thermo Scientific Emergo, Landsmeer, The Netherlands). The samples were sectioned at −15 °C and a thickness of 12 μm. The sections were supported during sectioning using double-sided tape (Tesa) and transferred and thaw-mounted on SuperFrost Plus microscopic glass slides. The samples were stored at −80 °C until further use.

Slides with tissue sections were dried in the desiccator for 35 min. Matrix application was performed using a TM-sprayer (HTX Technologies, Chapel Hill, NC). CHCA was dissolved in methanol:water (70:30) at a concentration of 5 mg/mL. Thirteen layers were sprayed with a drying time of 10 s between layers using a nozzle temperature of 30 °C, a flow rate of 0.12 mL/min, nozzle velocity of 1300 mm/min, track spacing of 1.5 mm, and CC pattern.

#### 2.5.2 MALDI-MSI

A 9.4T SolariX Fourier transform ion cyclotron resonance (FT-ICR) mass spectrometer (Bruker Daltonik GmbH, Bremen, Germany) was used for MALDI-MSI acquisition operated with FTMS control (version 2.2.0, Bruker). The pixel size was set to 50 μm with SmartWalk enabled with a width of 25 μm and grid increment of 5 μm. The laser focus was set to a minimum (<30 μm), the laser power was set at 25%, and 750 laser shots were fired per pixel at a frequency of 2000 Hz. 1 M data points were acquired for each pixel for a *m*/*z* range of 100.44 to 1200 in positive ionization mode. The lower cutoff was set to *m*/*z* 350 by setting the Q1 mass to 350 to reduce the intensity of the matrix peaks in the lower mass range. A data reduction factor of 95% was used to save the reduced profile spectra. FlexImaging (version 5.0, Bruker) was used for setting up regions of interest for acquisition. The calibration of the instrument was performed before each measurement using red phosphorus.

#### 2.5.3 MALDI-MSI Data Analysis

All measurements were imported into SCiLS Lab 2022a (SciLS GmbH, Bremen, Germany) using the centroided mass spectra. Annotations of bone and bone marrow were manually performed in QuPath (v0.2.3)^38^ and imported into SciLS. The number of pixels per region of interest per sample varied based on the sample size, which was taken into account in further data analysis. The annotations were limited to bone and bone marrow for methodological reasons. Peak picking was performed on the overview spectrum of all regions using mMass (Open Source Mass Spectrometry Tool, version 5.5.0).^39^ The distribution images of these *m*/*z* values were inspected to remove matrix clusters and background peaks. The obtained peak list including detected isotopes was used in further data analysis. The imzML files including only the peaks of the reduced peak list were exported without normalization per sample with separate files for the bone and bone marrow regions. These imzML files were converted into MATLAB compatible files using an in-house-written R-script. The in-house-built ChemomeTricks toolbox (version 2.71c)^40^ for MATLAB (version 2014a, The MathWorks, Natick, MA) was used to perform principal component analysis-linear discriminant analysis (PCA-LDA) after normalization and autoscaling for comparison of the Citr and Cont group for 3, 7, 14, and 28 DPO separately for bone and bone marrow based on the detected intensities of the different peaks. The resulting discriminant functions (DFs) showed the separation between the two groups based on lipid profiles, as a smaller overlap in these DFs indicates bigger differences in the overall lipid profiles of the corresponding groups. These lipid profiles were used for further analyses, as they provide information about the contribution of the lipids to the separation between the two groups. Quantitative lipid analysis was not performed because of technical limitations of the applied methodology.

#### 2.5.4 Lipid Identification

The *m*/*z* values of interest were selected based on the lipid profiles from the DFs for each time point. These *m*/*z* values were selected based on the 30 highest unscaled loadings for each sample per time point. Isotopic and low intensity peaks were removed from the lists with interesting *m*/*z* values, as detection is challenging after fragmentation. MS/MS analysis was used for the identification of the molecules using collision-induced dissociation (CID) fragmentation. MS/MS data were manually acquired on a SYNAPT HDMS G2-Si coupled with a prototype uMALDI source (Waters Corporation, Manchester, UK) operated with MassLynx (version 4.1, Waters), which has been described by Barré et al.^41^ The MS/MS measurements were performed in sensitivity mode with a scan rate of 1.0 s per scan. The instrument was calibrated with red phosphorus twice a day. The laser fluence was set to 250 and 300 arbitrary units on bone marrow and bone, respectively. The MS/MS isolation window was optimized per *m*/*z* value and was between 1 and 1.5 Da. The trap collision energy varied between 15 and 25 arbitrary units. Lipid identifications were performed using a combination of MassLynx (version 4.1), LipostarMSI (version 1.3.0, Molecular Horizon, Bettona, Italy),^42^ and database searches in ALEX^123^ lipid calculator.^43^ Automated and manual identification were compared and combined to prevent misidentification and only lipids with a high confidence identification were included.

### 2.6 Protein Analysis with LC-MS

#### 2.6.1 Protein Extraction

Pieces of bone tissue, consisting of bone and bone marrow, per animal were homogenized using a pestle and mortar on liquid nitrogen. At least 30 mg of sample (range 37 to 238.7 mg) was placed into an Eppendorf tube containing 250 μL of 5% SSA. Samples were stored at −80 °C until further use.

The homogenized sample was thawed and centrifuged. The SSA was removed and 500 μL of 5 M urea/50 mM ABC was added to the remaining pellet. The proteins from the bone pellet were dissolved by shortly sonicating in an ultrasonic bath and 10 min at 10 °C in a thermoshaker at 750 rpm. Undissolved particles and the bone pellets were removed, and the proteins in the solution were transferred to 3 kDa filters. The dissolved proteins were filtered three times using 5 M urea/50 mM ABC, and any remaining undissolved particles were removed. The protein concentration of each sample was determined using Bradford Protein Assay (Bio-Rad Laboratories, Hercules, CA) according to the manufacturer’s protocol by measuring the absorption at 595 nm (optical density). Samples were stored at −80 °C until further use.

#### 2.6.2 Protein Digestion

A total of 50 μg of protein was used per sample for the protein digestion. The sulfur bridges were broken with 20 mM DTT for 45 min at room temperature. The protein samples were alkylated with 40 mM IAM for 45 min at room temperature in the dark. The alkylation was terminated using 20 mM DTT for 45 min.

A mixture of trypsin and LysC was used for protein digestion and was added at a ratio of 1:25 (enzyme:protein). The proteins were digested for 2 h at 37 °C in a thermoshaker at 250 rpm. The urea concentration of the samples was reduced to 1 M by adding 50 mM ABC. Further digestion took place overnight at 37 °C in a thermoshaker at 250 rpm. The addition of FA with a final relative concentration of 1% was used to terminate the digestion. The peptide samples were centrifuged to remove any remaining particles and stored at −80 °C until analysis.

#### 2.6.3 LC-MS/MS

A LC-MS based bottom-up proteomics experiment was conducted on the obtained peptide samples. The peptide separation was performed on a Thermo Scientific Ultimate 3000 Rapid Separation UHPLC system (Dionex, Amsterdam, The Netherlands) equipped with a PepSep C18 analytical column (15 cm, ID 75 μm, 1.9 μm Reprosil, 120 Å). The samples were desalted on an online-installed C18 trapping column and were separated on the analytical column with a 90 min linear gradient from 5% to 35% ACN with 0.1% FA at a flow rate of 300 nL/min. The UHPLC system was coupled to an Orbitrap QExactive HF mass spectrometer (Thermo Scientific, GmbH, Bremen, Germany). Data-dependent acquisition (DDA) was used for the measurement of full MS and MS/MS spectra. The full MS scans were acquired for *m*/*z* 250–1250 at a resolution of 120 000 and were followed by MS/MS scans of the top 15 most intense ions at a resolution of 15 000.

#### 2.6.4 Data Analysis and Protein Identification

The DDA spectra were processed and analyzed with Proteome Discoverer (PD, version 2.2, Thermo Scientific) for the identification and quantification of the proteins. The search engine Sequest was used within the PD software applying the rat protein database (*Rattus norvegicus*, SwissProt TaxID = 10116, 8150 proteins included). The following settings were selected for the database search: trypsin as the enzyme (LysC has an overlapping cleavage site), a maximum of 2 missed cleavages, minimum peptide length of 6, precursor mass tolerance of 10 ppm, fragment mass tolerance of 0.02 Da, dynamic modifications of methionine oxidation and protein N-terminus acetylation, and static modification of cysteine carbamidomethylation. The default label-free quantification (LFQ) settings in PD were used for protein quantification. In short, the peptide precursor intensities were used for peptide abundancies. Normalization was performed on the total peptide amount. Protein ratios were calculated based on pairwise peptide ratios. Background-based ANOVA was applied for hypothesis testing.

One of the samples of Citr7 was excluded from the data analysis and LFQ after the initial data analysis. These analyses showed a much lower number (<40% compared to other samples) of proteins identified with high confidence in this sample. Data analysis was performed using the remaining five samples for Citr7 and all samples for the other groups. The number of identified proteins was a total of 1186 for all samples combined, of which 883 proteins were identified with high confidence (default PD settings, false discovery rate (FDR) threshold 1%). PCAs were performed to compare the protein profiles between the Citr and Cont groups per time point. The differentially expressed proteins for the Citr and Cont groups were determined for 3, 7, 14, and 28 DPO by protein ratio analyses to study the effect of citrulline supplementation. Proteins were considered as differentially expressed in one of the groups in the ratio with a fold change of 1.5 (log 2 of ≥0.58 or ≤−0.58) and an adjusted *p*-value of ≤0.05.

#### 2.6.5 Pathway Analysis

Pathway analysis was performed based on the differentially expressed proteins to identify the differentiating biological processes between the Citr and Cont groups per time point. Pathway analysis was performed using Reactome Pathway Database.^44^ The gene names related to the differentially expressed proteins were used for analysis. The *p*-value was set to ≤0.05 for the selection of involved pathways. In addition, the threshold for the FDR was set to ≤0.25 and to ≤0.05 for possible involved pathways and involved pathways with high confidence, respectively. In case multiple pathways that were connected in the pathway overview of Reactome were noticed to have the same matched gene name, one of these pathways was selected based on the specificity that could be expected based on the matched gene names.

## 3 Results

### 3.1 Comparison of Callus Formation and Biomechanical Strength

**Figure 3:**
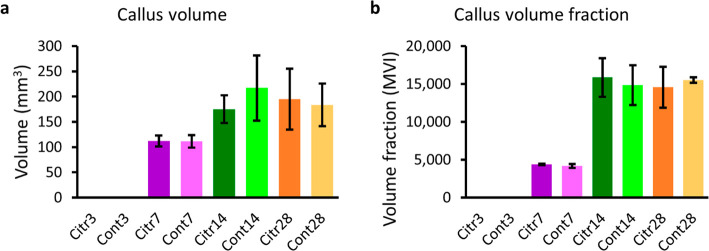
Comparison of (a) the fracture callus volume and (b) the fracture callus volume fraction for the citrulline supplementation (Citr) and control (Cont) group for the different time points (3, 7, 14, and 28 DPO). Abbreviations: MVI = mean voxel intensity.

None of the rats had a developed callus on the μCT images at 3 DPO in the Citr and Cont group. A fracture callus was present in all rats at 7 DPO and 14 DPO. Two rats in the Citr group and three rats in the Cont group no longer had a callus at 28 DPO, as these fractures healed. Example μCT images for each group are provided in Supporting Information Figure S1. The comparison of the fracture callus volume (Figure 3A) and volume fraction (Figure 3B) do not demonstrate significant differences between the Citr and Cont group. The bone volumes at 3 and 7 DPO are significantly higher in the Cont group than in the Citr group (Supporting Information Figure S2A). The bone volumes at 14 and 28 DPO and the bone volume fractions at all time points did not significantly differ (Supporting Information Figure S2A and S2B). The biomechanical strength at the fractured side did not show a significant difference between the Citr and Cont group at 42 DPO (Supporting Information Table S2).

### 3.2 Lipid Profile Comparison

#### 3.2.1 Lipid Profiles in Cortical Bone

**Figure 4:**
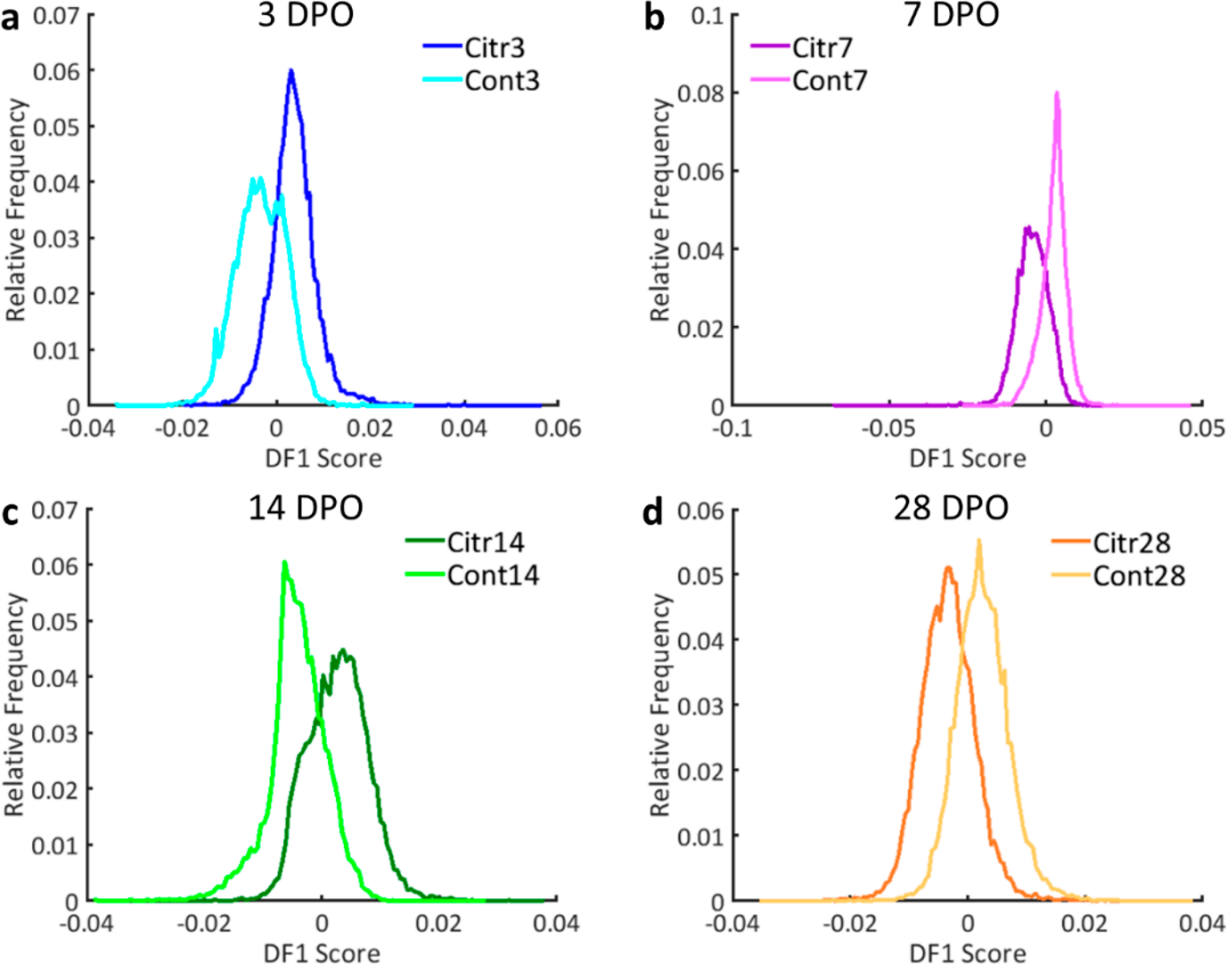
First discriminant function (DF1) for the lipid comparison of the citrulline supplementation (Citr) and control (Cont) group per time point for bone representing the results of the PCA-LDA analyses. DF1 plots are shown for (a) 3, (b) 7, (c) 14, and (d) 28 DPO representing 2.20, 1.47, 1.94, and 1.61% of the variance in the data set, respectively. The DF plots display how well the Citr and Cont groups can be separated based on the lipid profiles acquired from bone, showing the effect of citrulline supplementation on the lipid profiles.

The PCA-LDA analyses showed no complete separation for 3, 7, 14, and 28 DPO between the Citr and Cont group for bone, as represented in Figure 4A–D. The overlap in the discriminate distributions of the Citr and Cont group indicate the small differences in the lipid profiles between the treatment and control groups. Exemplary lipid distributions for the bone regions are provided in Supporting Information Figure S3. The major classes of identified lipids contributing to the differences between the groups are phosphatidylcholines (PCs) and lysophosphatidylcholines (LPCs) (see Supporting Information Table S3). Furthermore, two acyl carnitines (CARs, namely CAR 16:0 and CAR 18:1), triacyl/alkylglycerides (TGs), and a sphingomyelin (SM, namely SM 34:1;O2) contributed. The LPCs with an alkyl group, such as LPC O-16:2 and LPC O-18:2, are contributing more to the lipid profiles of the Citr group at different time points. On the contrary, TG 52:5 and TG 54:6 are only contributing to the Cont group at 28 DPO. The different lipids from the other lipid classes contribute to both the Citr and Cont groups and depend on the lipid and the time point. Interestingly, most of the lipids contributing to 3, 7, and 28 DPO overlap in the Citr group, while no overlap is seen for 14 DPO (Supporting Information Figure S4A). Quite a high number of lipids contributing to the Cont group display overlap with other time points; especially, overlap between 7 DPO with other time points can be noticed (Supporting Information Figure S4B). No lipid is contributing to separation at all time points in either the Citr or Cont group.

#### 3.2.2 Lipid Profiles in Bone Marrow

**Figure 5:**
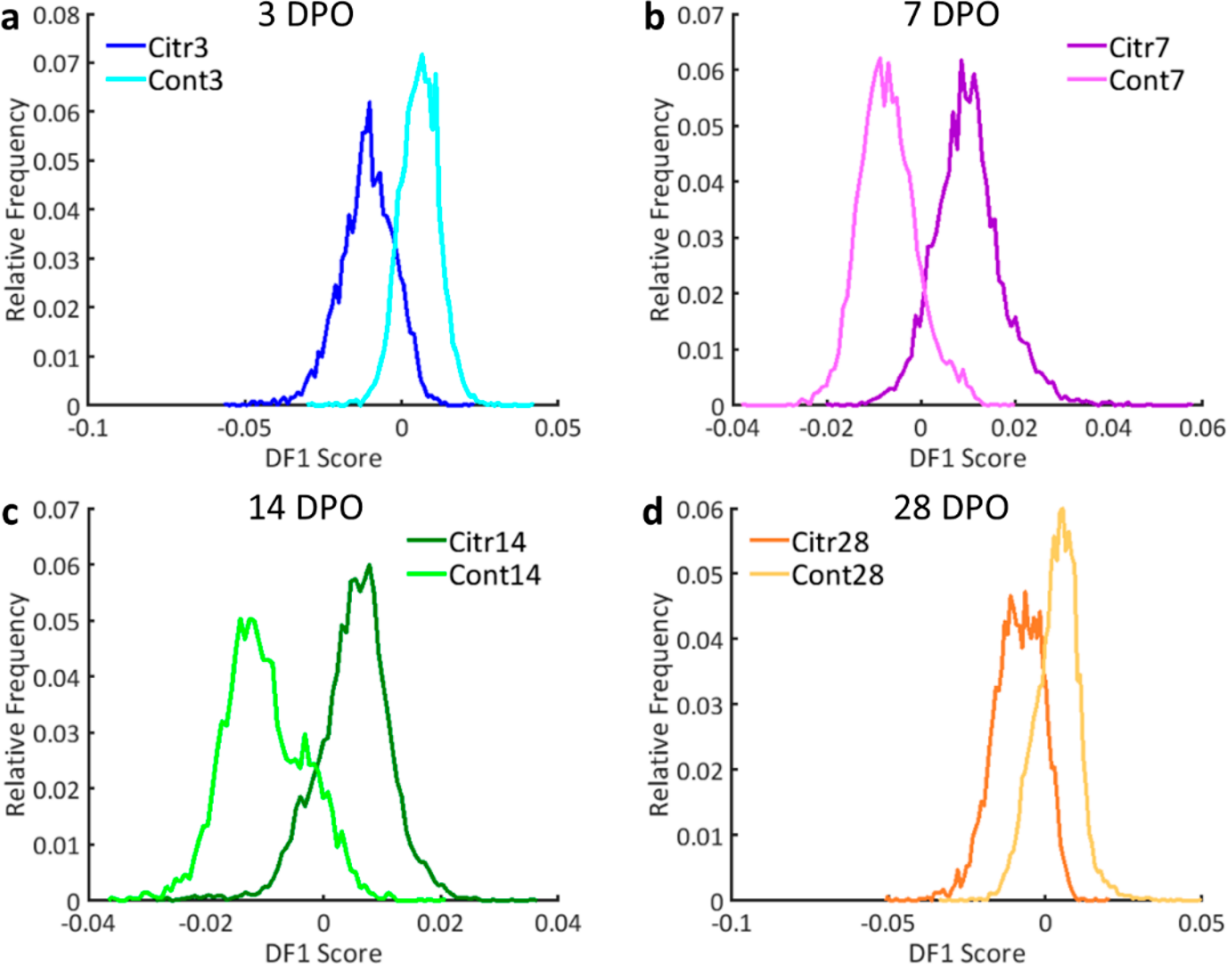
First discriminant function (DF1) for the lipid comparison of the citrulline supplementation (Citr) and control (Cont) group per time point for bone marrow representing the results of the PCA-LDA analyses. DF1 plots are shown for (a) 3, (b) 7, (c) 14, and (d) 28 DPO representing 2.69, 3.46, 3.53, and 2.75% of the variance in the data set, respectively. The DF plots display how well the Citr and Cont groups can be separated based on the lipid profiles acquired from bone marrow, showing the effect of citrulline supplementation on the lipid profiles.

Figure 5A, B, C, and D show the separation between the Citr and Cont group for bone marrow for 3, 7, 14, and 28 DPO, respectively, based on the PCA-LDA analyses. The separation between the Citr and Cont group is better for 7 and 14 DPO than for 3 and 28 DPO, as the overlap between the discriminate distributions of the Citr and Cont group is less. Therefore, the differences in the lipid profiles between the Citr and Cont groups at 7 and 14 DPO are probably bigger than at 3 and 28 DPO. Exemplary lipid distributions for the bone marrow regions are provided in Supporting Information Figure S5. PCs and LPCs are the major classes of identified lipids contributing to the separation between the Citr and Cont group at different time points, but also one CAR (namely CAR 16:0), one SM (namely SM 34:1;O2), and two TGs (namely TG 52:5 and TG 54:6) contributed (Supporting Information Table S4). The CAR and LPCs with an alkyl group, such as LPC O-16:2 and LPC O-18:2, are only contributing to the lipid profile of the Citr group, while the two TGs contribute to the Cont group, specifically at 28 DPO. The different LPCs and PCs seem to contribute to both groups in their separation at different time points. Interestingly, the LPCs are contributing mainly to the Citr group, while the PCs contribute more to the Cont group. Some lipids contribute to both the Citr and Cont groups, but their contribution to the lipid profile of each group is at different time points. Only overlap of three lipids between 3 and 14 DPO can be noticed for the Citr group (Supporting Information Figure S6A). More overlap in contributing lipids between the different time points can be seen for the Cont group, especially for the overlap of 3 DPO with different time points (Supporting Information Figure S6B). No lipid is contributing to the separation at all time points in either the Citr or Cont group.

The separation between the Citr and Cont groups is better for bone marrow than for bone when comparing the overlap of the DFs in Figures 3 and 4. A pattern of medium separation at 3 DPO, good separation at 7 and 14 DPO, and limited separation at 28 DPO is seen for bone marrow. However, this pattern was not present for cortical bone. The identified lipid classes overlap between bone and bone marrow, and the main classes are LPC and PC for both bone and bone marrow (Supporting Information Tables S3 and S4). Four, zero, three, and zero lipids overlap between bone and bone marrow for the Citr group for 3, 7, 14, and 28 DPO, respectively. Four, six, zero, and three lipids overlap between bone and bone marrow for the Cont group for 3, 7, 14, and 28 DPO, respectively.

### 3.3 Comparison of Proteins and Pathways

#### 3.3.1 Proteins in Cortical Bone and Bone Marrow

Protein profiles were obtained for each sample group from the mix of crushed cortical bone and bone marrow. The PCAs did not show a separation between the Citr and the Cont group for 3, 7, 14, or 28 DPO. PCA plots of the first two principal components (PC1 and PC2) are provided in Supporting Information Figure S7.

Ten, thirteen, nine, and five proteins were differentially expressed in the Citr group compared to the Cont group at 3, 7, 14, and 28 DPO, respectively (see volcano plots in Supporting Information Figure S8). Five, sixteen, nineteen, and nineteen proteins were differentially expressed in the Cont group compared to the Citr group at 3, 7, 14, and 28 DPO, respectively. An overview of the differentially expressed proteins can be found in Supporting Information Table S5. The overlap between proteins with higher abundance within the Citr and Cont group is limited for the different time points, except for 7 DPO (Supporting Information Figure S9). This can be attributed to the different fracture healing phases that occur at these different time points. The overlap of proteins between the different time points is higher for the Cont group than for the Citr group, especially for 28 DPO. Comparison of the differentially expressed proteins between the Citr and Cont group shows that only four proteins overlap between them, namely cathepsin G (Ctsg), guanine deaminase (Gda), proteasome subunit beta type-2 (Psmb2), and slit homolog 1 protein (Slit1). The abundance of these proteins was higher at a later time point in the Cont group than in the Citr group.

#### 3.3.2 Pathway Analysis

**Figure 6:**
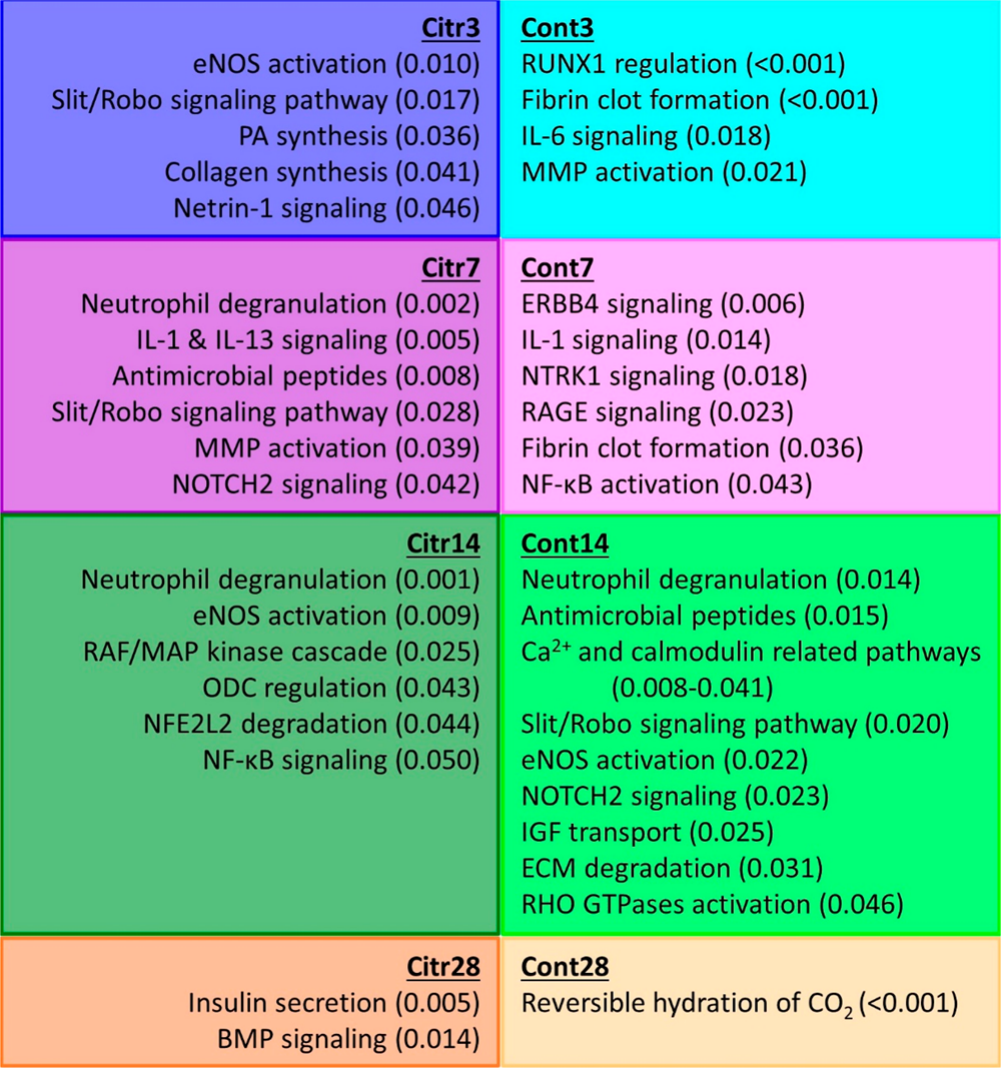
Selected identified pathways related to bone fracture healing for the citrulline supplementation (Citr) and control (Cont) group for 3, 7, 14, and 28 DPO with the related *p*-value in parentheses. Abbreviations: BMP = bone morphogenetic protein; ECM = extracellular matrix; eNOS = endothelial nitric oxide synthase = NOS3; ERBB4= erb-b2 receptor tyrosine kinase 4; IGF = insulin-like growth factor; IL = interleukin; MMP = matrix metalloproteinase; NFE2L = nuclear factor erythroid 2-related factor; NF-κB = nuclear factor kappa B; NOTCH = neurogenic locus notch homolog; NTRK = neurotrophic receptor tyrosine kinase; ODC = ornithine decarboxylase; PA = glycerophosphates; RAGE = receptor for advanced glycation end-products; RHO GTPases = RAS homolog family member of nucleotide guanosine triphosphate-ases; RUNX = runt-related transcription factor.

The more active pathways related to the differentially expressed proteins can be found in Supporting Information Table S6A–D for the different time points for the Citr and Cont groups. These pathways should be considered with caution, because of the low number of differentially expressed proteins on which they are based. Nevertheless, some of the identified pathways can be related to bone fracture healing, and these are displayed in Figure 6 for the Citr and Cont group per time point. The overlap between the pathways of the Citr group is limited, while the pathways of the Cont group show a higher number of overlapping pathways (Supporting Information Figure S10), despite the different fracture healing processes going on at the different time points.

## 4 Discussion

No significant differences in the fracture callus formation were observed between the Citr and Cont group based on the μCT data. MALDI-MSI analyses showed distinct lipid profiles that differed per time point in bone and bone marrow for both the Citr and Cont groups. Low numbers of differentially expressed proteins were identified in the Citr and Cont groups using LC-MS/MS. Pathway analyses showed different activated pathways for both the Citr and Cont groups at different time points and included pathways involved in the citrulline–arginine–nitric oxide metabolism. Molecular effects of citrulline supplementation on fracture healing are revealed by MS techniques, while μCT did not show any significant effect.

### 4.1 μCT Analyses: Fracture Callus Formation Displays No Significant Difference

The analyses of the μCT images did not show significant differences between the Citr and Cont group in the fracture callus volume and volume fraction (Figure 3). Our results are in line with the results of Rajfer et al., as they did not observe a significant difference in among others the callus volume at 14 and 42 DPO between a control group and a group that received a supplement including citrulline.^22^ On the contrary, Meesters et al. showed a significant difference in the callus volume between a citrulline supplementation and a control group at 14 DPO in mice.^16^ Direct comparison with these studies is complicated by methodological differences.

None of the rats showed callus formation at 3 DPO, which is expected in the inflammatory phase. The fracture callus volume and volume fraction were slightly, but insignificantly, higher in Citr7 than in Cont7. This could indicate that the callus formation in Citr7 is slightly further developed than in Cont7. The fracture callus volume was smaller and the volume fraction was higher in Citr14 compared to Cont14, although these were insignificant. This combination could indicate that the fracture healing process was further developed in the Citr group than in the Cont group, as more of the soft callus has been replaced by hard callus. There was no significant difference in the number of rats that healed at 28 DPO. The fracture callus volume was higher and the volume fraction was lower in Citr28 than Cont28 in the remaining rats. This could imply that the healing process was advanced less in the Citr group compared to the Cont group, which might be related to the end of citrulline supplementation at 14 DPO. These results could suggest a subtle positive effect of citrulline supplementation in the early phases of fracture healing, while this effect is no longer observed at 28 DPO.

### 4.2 Lipid Analyses: Identified Lipids Show Distinct Profiles throughout Fracture Healing

The separation between the Citr and Cont group was better for bone marrow than for bone, as shown in Figures 4 and 5. The best separations between the Citr and Cont group were for 7 and 14 DPO and slightly less at 3 DPO for bone marrow. This could indicate that the largest effect of the citrulline supplementation on the lipid profiles was during the soft and hard callus formation. The smaller difference between Citr28 and Cont28 could also be related to the ending of citrulline supplementation at 14 DPO. The better separations in bone marrow could indicate that the differences in lipid profiles between the Citr and Cont groups are bigger in bone marrow compared to bone, potentially relating to their higher importance during fracture healing. However, bone marrow contains a higher percentage of lipids than bone and the extraction of lipids from bone is more challenging than from bone marrow due to the mineralization of the tissue.^13^ These differences could reduce the desorption and ionization efficiency of bone in comparison to bone marrow.^45^ These experimental consequences should be similar between groups and, therefore, citrulline supplementation has the biggest effect on the lipid profiles from bone marrow at 3, 7, and 14 DPO.

The lists identified lipids that contribute to the separation between the Citr and Cont groups showed a great amount of overlap between bone and bone marrow (Supporting Information Tables S3 and S4). The changing effect of citrulline supplementation throughout fracture healing is demonstrated by the lack of overlap between time points and differences between bone and bone marrow and could indicate that different lipid patterns are involved in the fracture healing process in both tissues. In general, CARs and LPCs with an alkyl group contributed more to the Citr group, while TGs were specifically contributing to Cont28. The LPCs, PCs, and SM contributed to the Citr and Cont group depending on the time point and in different patterns between specific lipids. Overall, the lipid profiles of bone and bone marrow showed specific lipids contributing to the Citr and Cont group at different time points, as a result of the citrulline supplementation.

Unfortunately, the knowledge about the involvement and importance of lipids during fracture healing is limited and usually generalized per lipid class. Already in 1980, Boskey et al. showed that the ratio of different phospholipid classes insignificantly changed when comparing different time points during fracture healing.^46^ In addition, we recently published a paper describing different lipid profiles throughout fracture healing in the fracture hematoma.^47^ The observed lipid classes and most of the identified lipids overlap between our previous and current study, which signifies the importance of these classes and lipids during fracture healing. The observed lipid classes that contribute to the separation between the Citr and Cont group are CARs, LPCs, PCs, SM, and TGs. PCs are an essential source of lipid-derived secondary messengers and play a role in cellular metabolism as well as energy production.^13,48,49^ PCs are essential in the regulation of soft callus formation via the promotion of chondrocyte proliferation and differentiation as well as during the replacement of the soft callus by the hard callus via osteoblast proliferation and function.^50,51^ PCs containing a 20:5 fatty acid chain have been shown to promote osteoblast differentiation.^52^ Furthermore, PCs are potentially involved in the recruitment of osteoclasts.^50^ LPCs inhibit osteoclast formation, but promote osteoclast function.^47^ The dietary intake of choline, which is required for the synthesis of PCs, improved bone mineral density.^53^ SMs are an important source of ceramides and phosphocholine as well as secondary messengers.^13,54,55^ Impairment of the SM metabolism can result in abnormal cartilage development and reduction of bone mineralization.^13,54,55^ Furthermore, insufficient levels of SM can result in impaired bone development via the regulation of osteoblast differentiation and mineralization.^55^ TGs are commonly used for energy storage mainly in bone marrow.^13^ This matches well with the contribution of TGs only to Cont28, as during the bone remodeling phase also the energy storage is restored. Most of the observed lipid classes are involved during different fracture healing phases, and, therefore, differences in expression can be expected. However, the role of specific lipids from bone and/or bone marrow during fracture healing remains unknown and extrapolation to the effect of citrulline supplementation is impossible.

### 4.3 Protein Analyses: Citrulline Supplementation Has a Changing Effect throughout the Fracture Healing Process

The effect of citrulline supplementation on the protein profiles throughout fracture healing is expected to be quite subtle, as PCAs did not show different protein profiles for the Citr and Cont groups (Supporting Information Figure S7). This small effect was also reflected in the low numbers of differentially expressed proteins at the different time points. The highest numbers of differentially expressed proteins in the Citr group were seen at 3, 7, and 14 DPO. This could indicate a bigger effect of the citrulline supplementation during the earlier stages of the fracture healing, which matches with previous research that showed citrulline supplementation improved angiogenesis and callus formation.^16,22^ The limited overlap of the differentially expressed proteins between different time points in the Citr and Cont group can be related to the different fracture healing phases (Supporting Information Figure S9). Therefore, the effect of citrulline supplementation on the protein expression throughout the fracture healing process was not constant, indicating that citrulline supplementation could affect the process in different ways.

The differentially expressed proteins per sample group listed in Supporting Information Table S5 provide an overview, but a separate discussion of each protein is beyond the scope of this paper. Some molecules that are a part of the extracellular matrix (ECM) of bone tissue were differentially expressed throughout the fracture healing process in the Citr or Cont group. These included different collagens, fibromodulin, and thrombospondin, which have a role in the regulation of bone formation and resorption via effects on the osteoblast and osteoclast function.^12,56^ Besides, the synthesis of collagen can be affected by the citrulline supplementation via de production of polyamines (see Figure 1). This explains the differentially expressed collagen in the Citr3 group, while the differentially expressed collagen in the Cont14 group might be related to a relatively delayed collagen synthesis. Furthermore, different myosins, myosin light chains, and troponins were differentially expressed in the Cont group, while an actin and annexin were differentially expressed in the Citr group. The involvement of actin and different myosins during fracture healing is not surprising, due to their role in the cytoskeleton. Myosins have been shown to play an important role in osteoclast differentiation and function, while annexins are important in osteoblast differentiation and function.^57−59^ However, little is known about the role of these proteins and many other differentially expressed proteins during fracture healing.

Four proteins were differentially expressed in both the Citr and Cont group, although at different time points, namely cathepsin G (Ctsg), guanine deaminase (Gda), proteasome subunit beta type-2 (Psmb2), and slit homolog 1 protein (Slit1). These proteins are involved in many different pathways and do not have one general effect. An enhancement of the fracture healing process in the Citr group in comparison to the Cont group could be implied by the earlier time point of differentially expression in the Citr group of these four proteins.

### 4.4 Pathway Analyses: Citrulline Supplementation Results in Enhanced Fracture Healing

The more active pathways displayed in Figure 6 can be related to different processes during bone fracture healing. The combined effects of these pathways will be discussed here. An extensive description of these pathways and other activated pathways per time point is provided in Supporting Information S1. However, these results should be considered with caution, as the pathway analyses are based on low numbers of differentially expressed proteins.

Only one more active pathway is related to changes in lipid metabolism, namely synthesis of PA for the Citr3. The proteins related to lipid metabolism are not differentially expressed, because of the small differences in the lipids between Citr and Cont per time point. The lack of knowledge about lipids and their regulation by pathways during bone fracture healing hinders the further combined analysis of the identified lipids and more active pathways in this study.

Different pathways related to the citrulline–arginine–nitric oxide metabolism were activated at different time points, namely eNOS/NOS3 activation in Citr3 and Citr14 as well as Cont14. The regulation of ornithine decarboxylase (ODC) is more active in Citr14, which is important in the metabolism of polyamines. These activations can be related to the importance of nitric oxide in the inflammatory phase, fracture callus formation, and bone remodeling as well as the formation of collagen via polyamines (Figure 1).^16,18−21^

The inflammatory phase (3 DPO) of the Citr group was characterized by activation and regulation of inflammatory pathways, promotion of angiogenesis, and potentially more active start of the soft callus formation. Activation of inflammatory pathways and fibrin clot formation were more active processes in the Cont3. The more active processes in the Citr group during soft callus formation (7 DPO) resulted in cell recruitment, proliferation, and differentiation of especially chondrocytes and osteoblasts as well as soft callus formation, and promotion of angiogenesis. The more active processes in Cont7 partly overlapped with these, as the active pathways resulted in the proliferation and differentiation of chondrocytes and osteoblasts as well as soft callus formation, promotion of angiogenesis, fibrin clot formation, and activation of inflammatory pathways. The more active processes in the Citr group during the hard callus formation (14 DPO) were differentiation and regulation of function of osteoblasts and osteoclasts resulting in bone remodeling. Regulation of bone remodeling and hard callus formation by proliferation, differentiation, and regulation of function of osteoblasts and osteoclasts were also active processes in Cont14, while angiogenesis is also still active. Different activated pathways indicated an ongoing and active bone remodeling process as well as regulation of the energy metabolism in the Citr group during the bone remodeling phase (28 DPO). At the same time point, only bone remodeling processes were more active in the Cont group.

The combination of the activated pathways and resulting processes suggested a slightly enhanced fracture healing process in the Citr group compared to the Cont group. This difference was less pronounced during the bone remodeling phase, as a lower number of more active pathways were observed at 28 DPO. This could be related to the ending of citrulline supplementation after 14 DPO. The fracture healing process seemed to be enhanced via improved angiogenesis and earlier formation of the soft and hard callus as a result of the citrulline supplementation. These results were in agreement with the previous citrulline supplementation study performed in mice by Meesters et al.^16^ On the contrary, the inflammation phase and angiogenesis take longer in the control group.

### 4.5 Limitations and Future Research

This study applied different mass spectrometry techniques for the detection of lipids and proteins from bone tissue. Nevertheless, there are some methodological limitations that merit discussion. Lipid MSI was only performed in positive ionization mode, as we have previously shown that this ionization provides better results than the negative ionization mode.^27^ Acquisitions in only positive ion mode, will affect which lipid classes can be extracted.^60−62^ Mainly PCs and LPCs were detected in this study, but also some CARs, a SM, and two TGs were observed. We believe MALDI-MSI acquisition only in positive ionization mode provides sufficient information in this study, as the major lipid classes present in bone and bone marrow (TGs and PCs) are more commonly detected in this mode.^13^ However, additional information about other lipid classes can be provided by MALDI-MSI in negative ionization mode. The protein extractions were performed on crushed pieces of bone and, therefore, no separate analysis of bone and bone marrow proteins could be performed. The composition and function of bone and bone marrow are different.^63^ Separate analysis would have allowed to detect protein compositional changes for bone and bone marrow. The applied sample collection method resulted in differences in the ratio of bone and bone marrow. This might have affected the proteins detected and data analysis, although housekeeping proteins showed similar abundances between samples.

This study showed the effect of citrulline supplementation on lipids and proteins during fracture healing in a rat model. The observed effect of citrulline supplementation on lipid changes in bone and bone marrow should be confirmed in the future, as little is known about specific lipids during fracture healing. In addition, the role of the different lipid classes and specific lipids during fracture healing should be explored to improve understanding of the effect of citrulline supplementation. The observed differentially expressed proteins and the related activated pathways should be validated in further research. Especially, because of the low number of differentially expressed proteins and the high number of pathways with only a single matched gene name as a result hereof. Furthermore, it would be interesting to study the effect of citrulline supplementation in a nonunion animal model, as this supplementation seems to enhance fracture healing. Lastly, a clinical study would be necessary to see the effect of citrulline supplementation during bone fracture healing in human patients.

## 5 Conclusion

The effect of citrulline supplementation on fracture healing in a rat model was explored using different techniques. μCT analysis showed no significant differences in the fracture callus formation between the Citr and Cont group. Nevertheless, a slightly positive effect of citrulline supplementation on the fracture healing process could be observed at 7 and 14 DPO. Lipid analysis with MALDI-MSI showed distinct lipid profiles for the Citr and Cont groups for bone and bone marrow at the different time points. Mainly PCs and LPCs contributed to these different lipid profiles. Protein analysis with LC-MS/MS displayed different abundant proteins at the different time points in the Citr and Cont group, which indicated a changing effect of citrulline supplementation during the phases of fracture healing. The analysis of activated pathways indicated a slight enhancement of the fracture healing process in the Citr group via improved angiogenesis and earlier formation of the soft and hard callus. The differences between the Citr and Cont groups were smaller during the bone remodeling phase for the pathway analysis, which was also observed for the lipid profiles. Overall, citrulline supplementation resulted in a subtle positive effect on fracture callus formation, a distinct change in the lipid and protein profiles during the different fracture healing phases, and an enhancement of fracture healing based on pathway analysis. While μCT data did not show any significant differences, citrulline supplementation resulted in molecular changes in lipids, proteins, and pathways during bone fracture healing as shown with different MS techniques. So, mass spectrometry can be used to show molecular effects of a treatment in bone fracture healing, while effects could be missed with μCT analysis.

## Data Availability

The data sets generated and analyzed in the current study are available from the corresponding author upon reasonable request.

## Supporting Information Available

S1 - Pathway analysis per time point. Figure S1 - Example μCT images. Figure S2 - Comparison of the bone volume and density. Figure S3 - Exemplary lipid distributions for bone regions. Figure S4 - Venn diagrams of the lipids from bone. Figure S5 - Exemplary lipid distributions for bone marrow regions. Figure S6 - Venn diagrams of the lipids from bone marrow. Figure S7 - Principal component analyses of protein profiles of the citrulline supplementation and control groups. Figure S8 - Volcano plots of the differentially expressed proteins. Figure S9 - Venn diagrams of the differentially expressed proteins. Figure S10 - Venn diagrams of the more active pathways. Table S1 - Overview of sample group references and number of samples. Table S2 - Biomechanical testing results for the citrulline supplementation and control groups. Table S3 - Lipid assignments for bone. Table S4 - Lipid assignments for bone marro. Table S5 - Proteins with higher abundance for the citrulline supplementation and control groups. Table S6 - More active pathways for citrulline supplementation and control groups (PDF)
