# Supplementary material for: Mass Spectrometry Reveals Molecular Effects of Citrulline Supplementation during Bone Fracture Healing in a Rat Model
Source: J Am Soc Mass Spectrom. 2024 Apr 29;35(6):1184–96. doi: 10.1021/jasms.4c00028 (PMC11157653; doi:10.1021/jasms.4c00028)
Supplement: Supplementary file 1 — js4c00028_si_001.pdf [file js4c00028_si_001.pdf]

Supporting Information – Mass spectrometry reveals molecular effects of citrulline supplementation during bone fracture healing in a rat model

Authors: Sylvia P. Nauta[1,2], Johannes Greven[3], Martijn Hofman[4], Ronny Mohren[1], Dennis M. Meesters[5,6], Diana Möckel[7], Twan Lammers[7], Frank Hildebrand[3], Tiffany Porta Siegel[1], Eva Cuypers[1], Ron M.A. Heeren[1], Martijn Poeze[2,6,*]

1. Division of Imaging Mass Spectrometry, Maastricht MultiModal Molecular Imaging (M4i) Institute, Maastricht University, 6229ER Maastricht, the Netherlands

2. Division of Traumasurgery, Department of Surgery, Maastricht University Medical Center, 6229HX Maastricht, the Netherlands

3. Department of Orthopedics, Trauma and Reconstructive Surgery, University Hospital RWTH Aachen, 52074 Aachen, Germany

4. Center of Musculoskeletal Surgery, Bonifatius Hospital Lingen, 49808 Lingen, Germany

5. Department of Genetics & Cell Biology, Maastricht University, 6229ER Maastricht, the Netherlands

6. NUTRIM, School for Nutrition and Translational Research in Metabolism, Maastricht University, 6229ER Maastricht, the Netherlands

7. Department of Nanomedicine and Theranostics, Institute for Experimental Molecular Imaging, RWTH Aachen University Clinic, 52074 Aachen, Germany

* Corresponding author: Martijn Poeze (m.poeze@mumc.nl)
Division of Traumasurgery, Department of Surgery, Maastricht University Medical Center
P. Debyelaan 25
6202 AZ Maastricht
The Netherlands

Index

Index S-2

Supporting Information S1 – Pathway analysis per time point S-3

Supporting Figure S1 - Example µCT images S-12

Supporting Figure S2 – Comparison of the bone volume and density S-12

Supporting Figure S3 – Exemplary lipid distributions for bone regions S-13

Supporting Figure S4 – Venn diagrams of the lipids from bone S-14

Supporting Figure S5 – Exemplary lipid distributions for bone marrow regions S-15

Supporting Figure S6 – Venn diagrams of the lipids from bone marrow S-16

Supporting Figure S7 - Principal component analyses of protein profiles of the citrulline supplementation and control groups S-17

Supporting Figure S8 – Volcano plots of the differentially expressed proteins S-18

Supporting Figure S9 – Venn diagrams of the differentially expressed proteins S-19

Supporting Figure S10 - Venn diagrams of the more active pathways S-19

Supporting Table S1 – Overview of sample group references and number of samples S-20

Supporting Table S2 - Biomechanical testing results for the citrulline supplementation and control groups S-20

Supporting Table S3 – Lipid assignments for bone S-21

Supporting Table S4 - Lipid assignments for bone marrow S-23

Supporting Table S5 - Proteins with higher abundance for the citrulline supplementation and control groups S-25

Supporting Table S6 – More active pathways for citrulline supplementation and control groups S-29

References S-34

Supporting Information S1 - Pathway analysis per time point

This Supporting information provides an extensive description of the more active pathways (see Supporting Table S6) per time point with a focus on pathways with biological meaning in bone fracture healing. One of the reasons for this decision is the high number of more active pathways in the different sample groups, which is partly caused by the low number of differentially expressed proteins. For the same reason, only one matched gene name is found for most pathways. Therefore, the pathway analyses should be considered with caution and be confirmed in future research. The pathways are discussed individually or in sets of closely related pathways. However, there is a great amount of interplay between the different pathways and different pathways affect each other or are part of one larger signaling pathway in some cases. These interplays between the different pathways are of great importance in the fracture healing process.

3 days post-operative: inflammatory phase

One of the interesting more active pathways in the Citr group is eNOS activation (see Supporting Table S6A). eNOS is also known as NOS3 and its activation is important in the production of nitric oxide.[1, 2] Nitric oxide has an important role as a secondary messenger in inflammatory pathways, in increasing blood flow at the fracture site, and potentially in infection prevention.[3-6] The activation of eNOS at this phase does match previous research, as Corbett *et al*. (1999) showed increased eNOS expression 1 day after fracture.[4] However, Zhu *et al*. (2001, 2002) found a higher protein expression of iNOS(/NOS2) than eNOS at 4 days.[6, 7] Nevertheless, activation of eNOS is the only form of NOS that is incorporated in the Reactome Pathway Database for nitric oxide metabolism. A more active nitric oxide metabolism via activation of eNOS can directly be related to the citrulline supplementation of the rats. This is in line with previous research, in which citrulline supplementation resulted in enhanced fracture healing among others by improvement of the inflammatory response.[8] Furthermore, signaling by ROBO receptors and Netrin-1 is more active in the Citr group. The ROBO receptors are part of the Slit/Robo signaling pathway.[9, 10] This pathway has a regulatory role in inflammation due to its inhibition of inflammatory cell infiltration.[10] In addition, the Slit/Robo signaling pathway can promote angiogenesis, which is important for the regulation of bone formation and nutrients and other supplies.[9, 10] Netrin-1 has been shown to be involved in angiogenesis as well as differentiation of and interaction between osteoblasts and osteoclasts.[9] The higher activity of the signaling by ROBO receptors and Netrin-1 in the Citr group are connected and can potentially indicate faster healing in this group, due to the inhibition of inflammation and promotion of angiogenesis. Furthermore, pathways related to PA and collagen synthesis are more active in the Citr group. PA is important in the regulation of inflammation via the secretion of inflammatory cytokines, like interleukins (ILs).[11] Upregulation of iNOS expression by PA was shown in macrophages by Lim *et al*. (2003).[11] The activation of PA synthesis indicates an active

regulation of the inflammatory response and could potentially be related to the citrulline supplementation. Collagen has a critical role in fracture healing, also because of its essential role in the extracellular matrix (ECM) of bone and hydroxyapatite formation.[12, 13] Collagen formation during the inflammatory phase is essential for cell anchoring and in the formation of callus.[12, 13] Activation of this pathway in the Citr group could indicate an earlier start of the formation of the soft callus in comparison to the Cont group.

Two related more active pathways in the Cont group are the regulation of interleukin gene transcription by RUNX1 (runt-related transcription factor 1) and interleukin-6 (IL-6) signaling. IL-6 is one of the pro-inflammatory cytokines and promotes angiogenesis.[5, 14-18] ILs and other pro-inflammatory cytokines show a peak expression during the first days of fracture healing.[14, 18] Activation of the IL-6 signaling matches with its role in the fracture healing process, while higher activation in the Cont group could indicate a more active inflammatory response than in the Citr group. The activation of IL gene transcription by RUNX1 is probably related to IL-6 signaling, as these pathways have the same single matched gene name. Pathways related to fibrin clot formation are more active in the Cont group. The formation of a fibrin clot is the start of the fracture healing process and forms the hematoma, which is the scaffold for soft callus formation.[5, 16, 19] Higher activity of these pathways in the Cont group can indicate a faster completion of the clot formation in the Citr group compared to the Cont group. Furthermore, matrix metalloproteinases (MMPs) are activated in the Cont group. MMPs facilitate cell migration and the formation of blood vessels through the bone matrix, also by releasing growth factors from the remaining ECM.[12, 20] MMPs play a role in chondrocyte proliferation and differentiation.[20] Activation of MMPs in the Cont group could indicate a slower angiogenesis and chondrocyte differentiation in comparison to the Citr group.

7 days post-operative: soft callus formation

One of the pathways more active in the citrulline supplement group at 7 days post-operative (DPO) is glycogen breakdown (see Supporting Table S6B). Glycogen breakdown results in glucose 1-phosphate and is important in glucose homeostasis, which can enter glycolysis for energy production.[21] However, the glycolysis pathway was not more active in the Citr group in comparison to the control group. Higher activation of the glycogen breakdown in the Citr group could indicate a higher need for glucose related to, for example, cell differentiation in comparison to the Cont group. Neutrophil degranulation is more active in the Citr group. Neutrophils are the first inflammatory cells that arrive at the damaged tissue site and their number decreases after the first days.[19, 22, 23] Neutrophils presence increases again in later phases of fracture healing in blood and their presence is related to normal fracture healing.[22] Neutrophils release the contents of their granules during degranulation.[23] The importance of neutrophil presence and degranulation during later stages of fracture healing is not clear. Antimicrobial

peptides show a higher activity in the Citr group. Antimicrobial peptides are an important part of the innate immune system and can be produced by, among others, neutrophils.[23, 24] Besides this role, antimicrobial peptides can promote wound healing via modulation of cytokine production, cell migration and proliferation, angiogenesis, and ECM production, especially collagen.[24] Antimicrobial peptide could potentially have a similar role in fracture healing, because of the similarities in early phases of wound and fracture healing. The antimicrobial peptides could indicate a higher activity of cell proliferation, ECM formation, and angiogenesis during the soft callus formation in the Citr group in comparison to the Cont group. Different signaling pathways are more active in the Citr group, namely multiple interleukins, including IL-1 and IL-13, NOTCH2 (neurogenic locus notch homolog 2), and ROBO receptors. IL-1 promotes the formation of the soft callus by the recruitment of cells and differentiation of osteoblasts and osteoclasts as well as the promotion of angiogenesis.[14, 16-18] IL-13 enhances osteoblast differentiation and function resulting in bone formation.[16] Based on these functions the activation of IL signaling matches well with the fracture healing phase. NOTCH signaling pathways have different functions during fracture healing that can be contradicting and are depending on cell and receptor-ligand combination.[25-27] On one hand, these pathways are important for the promotion of the proliferation of progenitor cells during the early stage of osteoblast formation, but can inhibit osteoblast differentiation.[26, 27] At the other hand, NOTCH2 enhances osteoclast formation and activation emphasizing its double role in fracture healing.[25-27] Furthermore, NOTCH signaling pathways have a critical role in angiogenesis during bone development.[25-27] Higher activity of NOTCH2 at this stage can be related to the proliferation of osteoblast progenitor cells. The Slit/Robo signaling pathway regulates bone formation and resorption, especially soft callus formation by promoting chondrocyte differentiation via the inhibition of β-catenin in the Wnt pathway.[9] In addition, the Slit/Robo signaling pathway can promote angiogenesis, which is an important event occurring during soft callus formation.[9] Higher activity of the Slit/Robo signaling pathway in the Citr group can indicate more soft callus formation and angiogenesis in comparison to the Cont group. Two pathways related to the transport of small molecules are more active in the Citr group, namely bicarbonate transporters and O_2/CO_2 exchange in erythrocytes. The more active pathway of reversible hydration of CO_2 in the Cont group is related to these pathways. Erythrocytes are important for the transport and exchange of the waste CO_2 for O_2.[28] Bicarbonate equilibrates the production of CO_2, as reversible hydration of CO_2 results in the production of bicarbonate and protons via carbonic anhydrases.[28] In addition, bicarbonate transporters have a role in pH-regulation during acid secretion occurring during the formation of hydroxyapatite during fracture healing.[28] Interpretation of the higher activation of these pathways is challenging due to the limited knowledge in the fracture healing processes. MMPs are more activated in the Citr group. MMPs play a role in angiogenesis, and osteoblasts and osteoclast recruitment and functioning, which is important in the replacement of the soft callus by a hard callus

and bone remodeling.[20] Higher activity in the Citr group could indicate more angiogenesis and recruitment of osteoblasts and osteoclasts, which can suggest an earlier start of the soft callus remodeling into the hard callus compared to the Cont group.

The metabolism of angiotensinogen to angiotensins is activated in both the Citr and Cont group, although the matched gene name is different. Angiotensinogen and angiotensins are part of the renin-angiotensin-aldosterone system (RAAS), which affects bone metabolism via activation of osteoclasts and inhibition of osteoblasts.[29] Expression of components of the RAAS has been shown in mature chondrocytes and osteoblasts during callus formation.[30] This matches with the activation of this pathway during soft callus formation in both the Citr and Cont groups, although the exact role is poorly understood.

Different signaling pathways are more active in the Cont group, namely IL-1, ERBB4 (erb-b2 receptor tyrosine kinase 4), and NTRK1 (neurotrophic receptor tyrosine kinase 1, TRKA). IL-1 is one of the interleukins that is also activated in the Citr group and, therefore, its role has been described above. Activation of IL-1 can be related to its role in angiogenesis and soft callus formation and potentially in callus remodeling in the Cont group. ERBB4 is a tyrosine kinase receptor for different angiogenic factors, which is related to the epidermal growth factor receptor (EGFR).[31] ERBB4 plays a role in cell migration and angiogenesis.[31] NTRK1 is a tyrosine kinase receptor for nerve growth factor (NGF) and is expressed in, among others, bone.[32] NTRK1 has been suggested to play a role in the proliferation and differentiation of chondrocytes and osteoblasts as well as to potentially promote angiogenesis.[32] Activation of ERBB4 and NTRK1 signaling in the Cont group can indicate delayed angiogenesis and chondrogenesis compared to the Citr group. Advanced glycosylation end-product receptor signaling and TRAF6 (TNF receptor associated factor 6) mediated NF-κB (nuclear factor kappa B) activation are two related pathways in the Cont group that show higher activation. Receptor for advanced glycation end-products (RAGE) is one of the receptors regulating the immune response and is involved in bone metabolism via osteoblast and osteoclast differentiation and function.[33] The NF-κB signaling pathway can be stimulated after activation of RAGE resulting in the secretion of pro-inflammatory cytokines.[33, 34] Besides, the TRAF6 mediated activation of NF-κB is important in the osteoclast differentiation and function.[33-35] Higher activation of these pathways could indicate that the inflammation phase is not completely finalized in the Cont group. On the other hand, it can indicate differentiation of osteoclasts, although these cells are usually not active during this phase. One of the fibrin clot formation pathways is more active in the Cont group. Fibrin clot formation is part of the first phase of the fracture healing process.[5, 16, 19] The activation of this pathway could indicate that this process is still ongoing or the remaining presence of a fibrin clot, which could imply a delayed fracture healing process in the Cont group in comparison to the Citr group.

14 days post-operative: hard callus formation

Interestingly, the eNOS (NOS3) is activated in both the Citr and Cont group at 14 DPO, while in the Citr group the regulation of ornithine decarboxylase (ODC) is also activated (see Supporting Table S6C). Nitric oxide production by NOSs is important in the regulation of fracture callus and bone remodeling, because of its effect on the proliferation, differentiation, and functioning of osteoblasts and osteoclasts.[3, 4, 6-8, 36] The eNOS activation at this time point for both groups matches with the eNOS mRNA found at 15 days by Diwan *et al*. (2000) and peak protein levels of eNOS at 14 days by Zhu *et al*. (2001, 2002).[1, 6, 7] Rajfer *et al*. (2017) demonstrated an increased iNOS expression during fracture healing at 14 days for COMB-4 supplement group, which included citrulline, in comparison to the Cont group.[37] Nevertheless, the pathways of iNOS are not included in the Reactome Pathway Database. The activation of eNOS in both the Citr and Cont group can be related to the stage of fracture healing. The matched gene names are different between the groups. The regulation of ODC is directly related to the metabolism of polyamines. Polyamines are a precursor of in the formation of collagen and are related to the arginine-citrulline-nitric oxide metabolism via the formation of ornithine.[8] The activation of the regulation of ODC might be related to the citrulline supplementation of the rats in this group, as more citrulline is available that can be converted to ornithine via arginine (see Figure 1). Tetrahydrobiopterin (BH4) metabolism is more active in the Cont group. BH4 is important in the regulation of NOS activity as a necessary cofactor.[38] Based on this function and the same matched gene name, the activation of this pathway is related to the eNOS activation in the Cont group at this time point. Another pathway that is activated in both the Citr and Cont group is neutrophil degranulation. Neutrophil degranulation results in the release of, among others, proteases and cytokines.[23] However, the importance and role of this process in fracture healing is poorly understood, as indicated above.

The metabolisms of different carbohydrates are activated in the Citr group, namely glycogen, glucose, and galactose, of which the glucose and glycogen metabolisms are activated in the Cont group as well. These different carbohydrates can be metabolized from the other carbohydrates and, therefore, are related.[21] These carbohydrates are important in the energy production, but also are involved in the production of, among others, fatty acids, glycolipids, and proteoglycans.[21] Further interpretation of these activated carbohydrate pathways is irrelevant, as most of them are activated in both the Citr and Cont group.

The RAF/MAP kinase cascade and NF-κB signaling are more active in the Citr group. The mitogen-activated protein (MAP) kinase signaling pathway has a wide range of biological effects and plays a regulatory role in bone development and homeostasis due to the regulation and stimulation of differentiation and maturation of osteoblasts as well as osteoclasts.[35] The activation of NF-κB is also involved in these effects.[34, 35] Activation of the RAF/MAP kinase cascade and NF-κB signaling matches

with the hard callus formation at this time point, due to their role in osteoblast and osteoclast differentiation and function. Especially, the activation of NF-κB could indicate early osteoclast activation for bone remodeling before the start of this phase in the Cont group. Furthermore, Hou *et al.* (2009) demonstrated increased iNOS expression via RAF/MAPK and NF-κB signaling pathways in ultrasound stimulated pre-osteoblasts.[39] Therefore, the activation of these pathways might be directly related to citrulline supplementation, also in relation to the eNOS activation. NFE2L2 (nuclear factor erythroid 2-related factor 2, NRF2) degradation is a more active pathway in the Citr group. NFE2L2 plays a role in different fracture healing phases as a key regulator of antioxidant enzyme expression and has been suggested to play a role in fracture callus formation via chondrocyte and osteoblast differentiation, although its role is poorly understood.[40] NFE2L2 degradation at this time point can be related to this role, as its higher activity in the Citr group can indicate earlier completion of osteoblast differentiation compared to the Cont group. Degradation of AXIN and Disheveled (DVL) is more active in the Citr group. AXIN and DVL both play a role in the Wnt/β-catenin signaling pathway, which is important in the differentiation of mesenchymal stem cells (MSCs) into osteoblasts or chondrocytes.[41, 42] AXIN is part of a multi-protein complex that phosphorylates β-catenin in the absence of Wnt ligands, which results in degradation.[41, 42] DVL is an intercellular protein that transduces the signal after binding of Wnt to its receptor and results in the accumulation of β-catenin that is followed by increased gene transcription.[41, 42] Activation of the degradation of both AXIN and DVL is unexpected due to their opposite roles. However, this is caused by the same single matched gene name for both pathways. Degradation of AXIN would be expected at this time point in the fracture healing process, as this would result in enhanced Wnt signaling via DVL resulting in increased osteoblast differentiation. Furthermore, negative regulation of NOTCH4 signaling was more active in the Citr group. The effects of NOTCH4 signaling on osteoblasts and osteoclasts as well as its role during fracture healing are unclear, due to the low expression levels in bone.[25, 26] The activation of this pathway can currently not be linked to bone fracture healing, and further research is necessary.

The signaling by ROBO receptors is a more active pathway in the Cont group. The Slit/Robo signaling pathway regulates bone remodeling via inhibition of osteoclasts formation and function as well as stimulating osteoblast migration and proliferation and, therefore, plays an important role in the regulation of bone formation and resorption.[9] In addition, the protein Robo-1 was differentially expressed in the Cont group at this time point. This protein is probably also involved in the Slit/Robo signaling pathway, but the gene name did not match with the Reactome database. The Robo-1 protein has been shown to play a role in bone homeostasis and remodeling.[43] Activation of the signaling by ROBO receptors pathway is related to the replacement of the soft callus with the hard callus. The higher activation of this signaling in the Cont group is later than in the Citr group could potentially

indicate a slower healing process in the Cont group compared to the Citr group. Different calmodulin induced events are more active in the Cont group, namely Cam-PDE1 activation, CaMK IV-mediated phosphorylation of CREB, and PKA activation. There are seven more active pathways in the Cont group related to the activation and regulation of NMDA receptors, which is an ion channel for among others Ca^{2+}. Also, the activation of the Ca-permeable Kainate receptor, sodium/calcium exchange, and reduction of cytosolic Ca^{2+} levels are more active pathways in the Cont group. All these different pathways are related to calcium ions and/or calmodulin. Calmodulin mediates many of the cellular effects of Ca^{2+} and one of the target proteins of Ca^{2+}/calmodulin is calcineurin.[44, 45] The signaling of calcium/calmodulin via calcineurin is also more active in the Cont group via two pathways. Ca^{2+}, calmodulin, and calcineurin play an important role in the proliferation and differentiation of osteoblasts.[45] Ca^{2+}, calmodulin, and calcineurin stimulate osteoclast differentiation as well, while changes in Ca^{2+} regulate bone resorption in mature osteoclasts.[44] Higher activation of these calcium-related pathways in the Cont group could indicate a later differentiation of osteoblasts and osteoclasts, which are needed for hard callus formation and bone remodeling, in comparison to the Citr group. Antimicrobial peptides is another pathway with a higher activity in the Cont group. Antimicrobial peptides can be generated by neutrophils.[23, 24] Because of this reason and the overlap in matched gene names, the activity of this pathway might be related to the activation of neutrophil degranulation. In addition, antimicrobial peptides might play a role in fracture healing via cell proliferation and ECM production, as their role in the promotion of wound healing has already been shown.[24] The higher activation of this pathway in the Cont group is at a later time point than in the Citr group, which could indicate a slower fracture healing process in the Cont group. NOTCH2 regulation of transcription is more active in the Cont group. The NOTCH signaling pathways have different target genes, including Hes1, which is involved in bone remodeling and regulation of bone mass.[25, 26] Activation of the NOTCH2 regulation of transcription can result in increased bone resorption.[25, 26] Bone resorption is important during the formation of the hard callus, but the formation of new bone is also important. Activation of this pathway in the Cont group can indicate more bone resorption in this group compared to the Citr group. The regulation of IGF transport and uptake of IGFBPs is more active in the Cont group. IGFs promote bone matrix formation during fracture healing via osteoblast differentiation and their actions are modulated by IGF-binding proteins (IGFBPs).[14, 18] The higher activity of the regulation of IGF transport and uptake of IGFBPs matches with the time point in the fracture healing process, but could indicate a delay in bone matrix formation in the Cont group in comparison to the Citr group. Degradation of the ECM is another pathway with higher activity in the Cont group. The degradation of ECM is mainly performed by MMPs.[20] This process is important during the replacement of the soft callus by the hard callus. Activation of ECM degradation in the Cont group could indicate slower healing in this group than in the Citr group. Another more active pathway is the metabolism of

angiotensinogen to angiotensins in the Cont group. Angiotensinogen and angiotensins activate osteoclasts and inhibit osteoblasts via de RAAS, and are expressed by osteoblasts, as indicated.[29, 30] The activation of this pathway can be matched with this role, as bone remodeling starts at the end of the hard callus formation. The longer activation of this pathway in the Cont group in comparison to the Citr group could indicate higher amounts of callus resorption in the Cont group. VEGFR2 mediated cell proliferation is more active in the Cont group. Vascular endothelial growth factor (VEGF) is important in the formation of blood vessels and is expressed by osteoblasts and chondrocytes.[14, 17, 18] Angiogenesis starts already during the inflammation phase via the angiopoietin-dependent pathway, while VEGF-mediated angiogenesis occurs at during the hard callus formation.[14, 17-19] Activation of this pathway can indicate that the angiogenesis in the Cont group is behind in comparison to the Citr group. More activity of the pathways related to RHO GTPases (RAS homolog family member of nucleotide guanosine triphosphate-ases) results in the activation of PAKs and NADPH oxidases in the Cont group. Activation of PAKs via GTPases plays an important part in the cytoskeletal reorganization and promotes bone resorption by osteoclasts.[46, 47] NADPH oxidases are important in the controlled formation of reactive oxygen species (ROSs) and ROSs can contribute to osteoblast and osteoclast differentiation.[48] Activation of these pathways can be related to the formation of the hard callus and the start of bone remodeling, in which both osteoblasts and osteoclasts are active. Higher activity of these pathways in the Cont group can indicate delayed osteoblast and osteoclast differentiation in comparison to the Citr group.

28 days post-operative: bone remodeling

Regulation of insulin secretion by acetylcholine is more active in the Citr group (see Supporting Table S6D). One of the functions of bone is its contribution to regulation of the energy metabolism.[49, 50] Especially, osteoblasts are involved in the regulation of the secretion of insulin, which might be related to the constant bone remodeling.[49, 50] The higher activation of this pathway in the Citr group could indicate higher activation of the bone remodeling pathways compared to the Cont group. Signaling by BMP (bone morphogenetic protein) is more active in the Citr group. BMPs are an important class of molecules involved in different phases of fracture healing.[17] Different BMPs have been shown to be upregulated during the bone remodeling phase of fracture healing, which indicates their involvement in this phase.[14, 17, 18] Higher activity of the signaling by BMPs in the Citr group in comparison to the Cont group could indicate a higher amount of ongoing bone remodeling.

Reversible hydration of CO_2 is more active in the Cont group. This process results in the production of bicarbonate, which can be equilibrated in an acidic environment.[28] An acidic environment is created by osteoclasts during bone resorption.[28] The higher activity of the reversible hydration of CO_2 in the Cont group could indicate a higher osteoclast activity and a less complete bone remodeling phase in

comparison to the Citr group. Purine catabolism is another pathway with higher activity in the Cont group. Bone remodeling and the functioning of osteoblasts and osteoclasts are mediated by extracellular purines, while their effect is highly dependent on the activated receptors.[51] Nevertheless, the role of purine catabolism during bone remodeling is unclear.

Supporting Figure S1 - Example µCT images

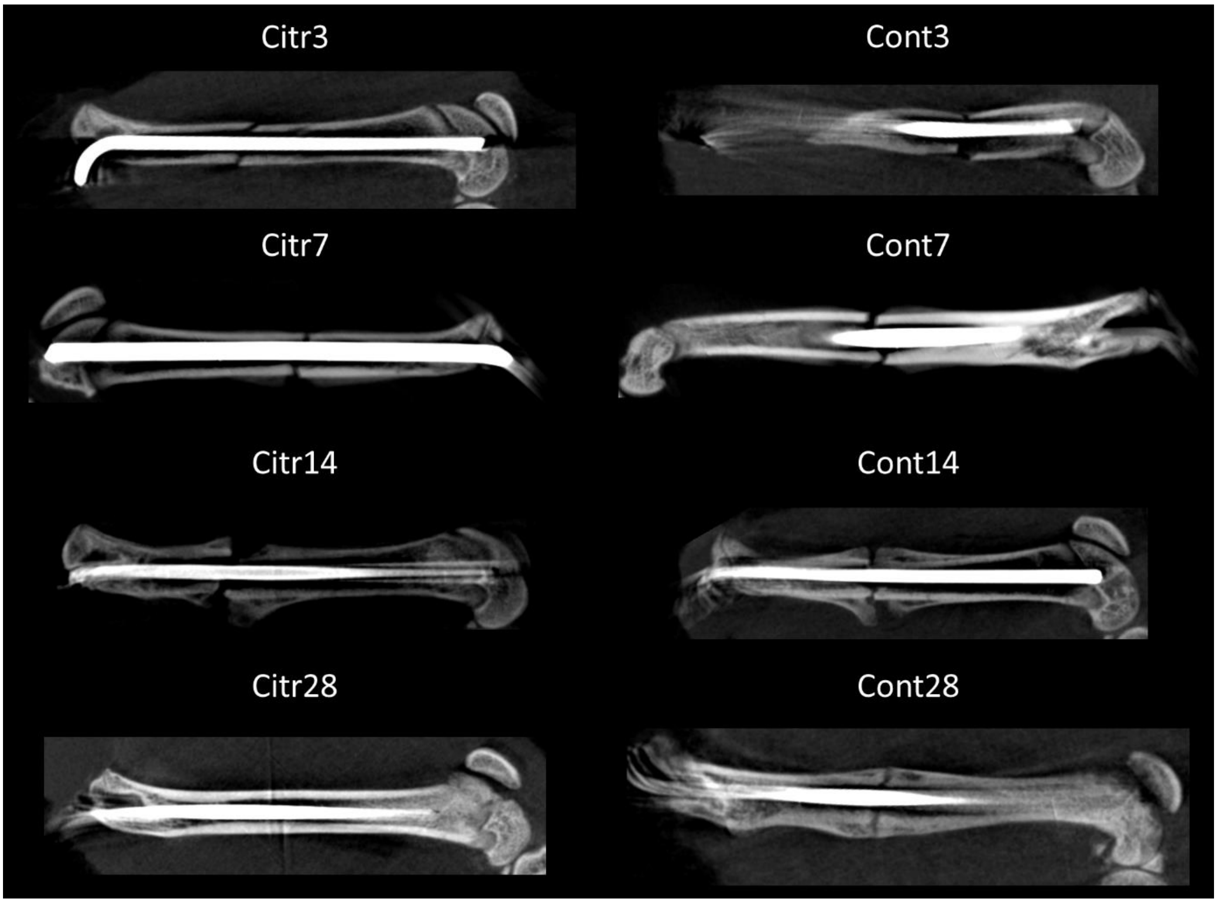

Supporting Figure S1: Example µCT images from one rat for each time point (3, 7, 14, and 28 DPO) for the citrulline supplementation (Citr) and control (Cont) group.

Supporting Figure S2 - Comparison of the bone volume and density

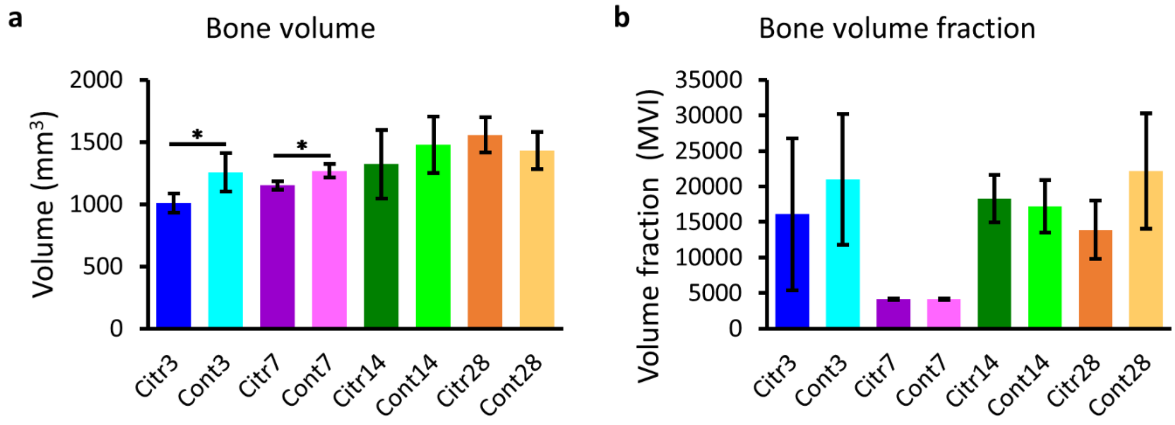

Supporting Figure S2: Comparison of **a** the bone volume and **b** the bone volume fraction for the citrulline supplementation (Citr) and control (Cont) group for the different time points (3, 7, 14, and 28 DPO). MVI = Mean Voxel Intensity. * indicates a significant difference with a p-value <0.01.

Supporting Figure S3 – Exemplary lipid distributions for bone regions

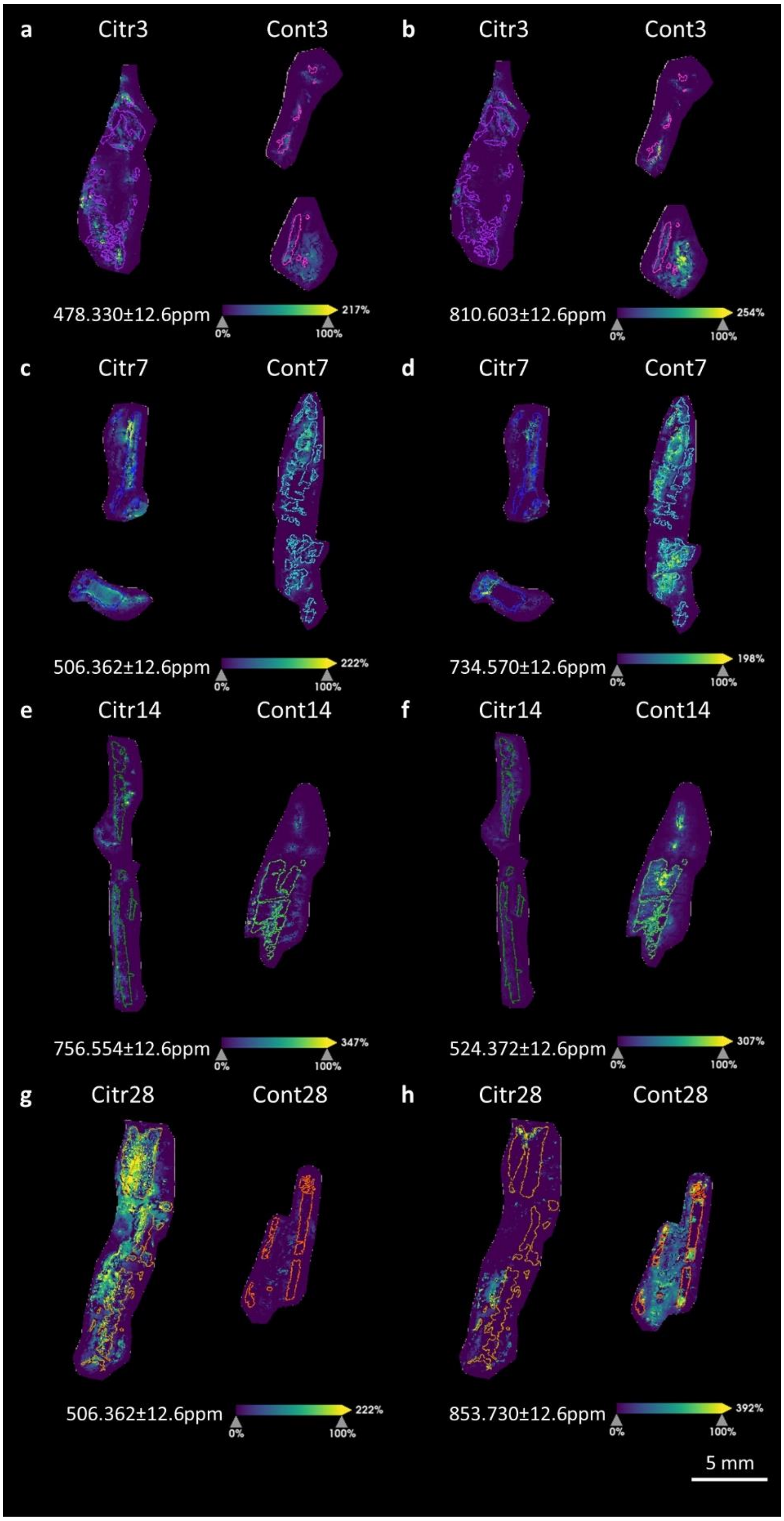

Supporting Figure S3: Exemplary lipid distributions for bone regions at different time points (3, 7, 14, and 28 DPO) for citrulline supplementation (Citr) and control (Cont) group for one sample per group,

as obtained with MALDI-MSI. The bone regions are enclosed by the colored lines. **a** *m/z* 478.330 contributing more to Citr3. **b** *m/z* 810.603 contributing more to Cont3. **c** *m/z* 506.362 contributing more to Citr7. **d** *m/z* 734.570 contributing more to Cont7. **e** *m/z* 756.554 contributing more to Citr14. **f** *m/z* 524.372 contributing more to Cont14. **g** *m/z* 506.362 contributing more to Citr28. **h** *m/z* 853.730 contributing more to Cont28. Identifications can be found in Supporting Table S3.

Supporting Figure S4 - Venn diagrams of the lipids from bone

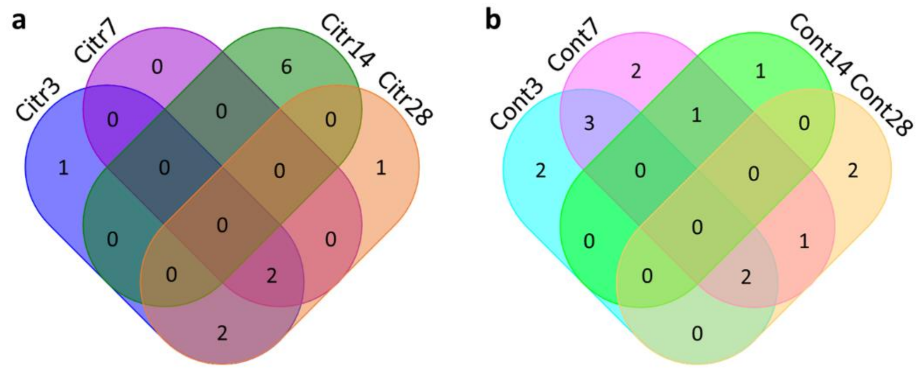

Supporting Figure S4: Venn diagrams of the lipids from bone contributing at the different time points (3, 7, 14, and 28 DPO) for citrulline supplementation (Citr) and control (Cont) group. Lipids detected with different adducts were grouped together per sample group. **a** Overlap in the contributing lipids in the Citr group. **b** Overlap in the contributing lipids in the Cont group. The numbers depicted in the overlapping areas indicate the contribution of a lipid to all the related time points.

Supporting Figure S5 – Exemplary lipid distributions for bone marrow regions

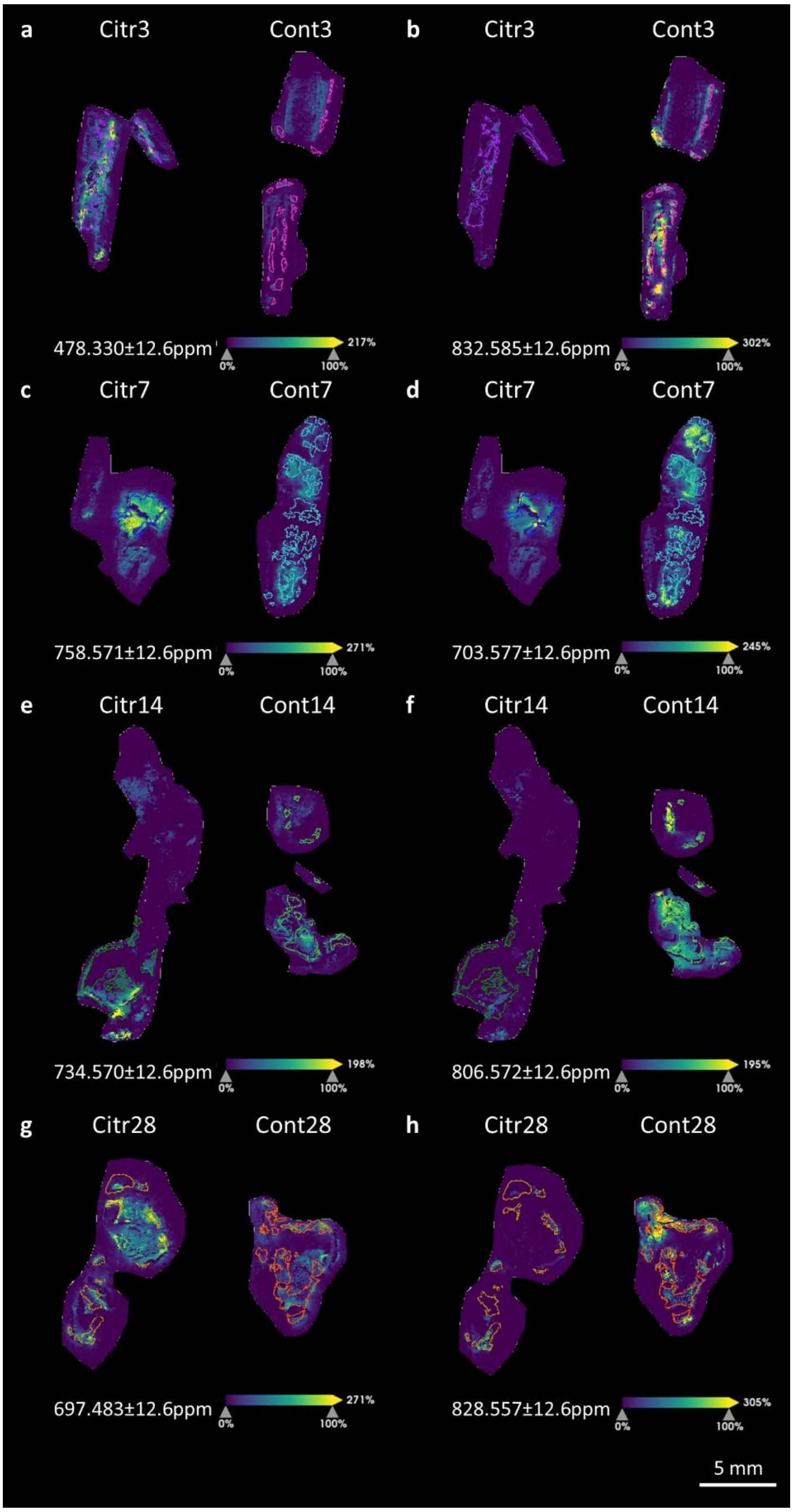

Supporting Figure S5: Exemplary lipid distributions for bone marrow regions at different time points (3, 7, 14, and 28 DPO) for citrulline supplementation (Citr) and control (Cont) group for one sample per group, as obtained with MALDI-MSI. The bone marrow regions are enclosed by colored lines. **a** *m/z*

478.330 contributing more to Citr3. **b** *m/z* 832.585 contributing more to Cont3. **c** *m/z* 758.571 contributing more to Citr7. **d** *m/z* 703.577 contributing more to Cont7. **e** *m/z* 734.570 contributing more to Citr14. **f** *m/z* 806.572 contributing more to Cont14. **g** *m/z* 697.483 contributing more to Citr28. **h** *m/z* 828.557 contributing more to Cont28. Identifications can be found in Supporting Table S4.

Supporting Figure S6 - Venn diagrams of the lipids from bone marrow

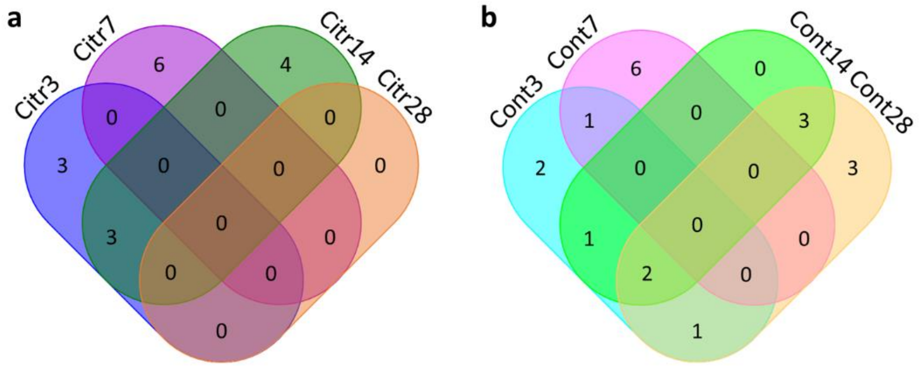

Supporting Figure S6: Venn diagrams of the lipids from bone marrow contributing at the different time points (3, 7, 14, and 28 DPO) for citrulline supplementation (Citr) and control (Cont) group. Lipids detected with different adducts were grouped together per sample group. **a** Overlap in the contributing lipids in the Citr group. **b** Overlap in the contributing lipids in the Cont group. The numbers depicted in the overlapping areas indicate the contribution of a lipid to all the related time points.

Supporting Figure S7 - Principal component analyses of protein profiles of the citrulline supplementation and control groups

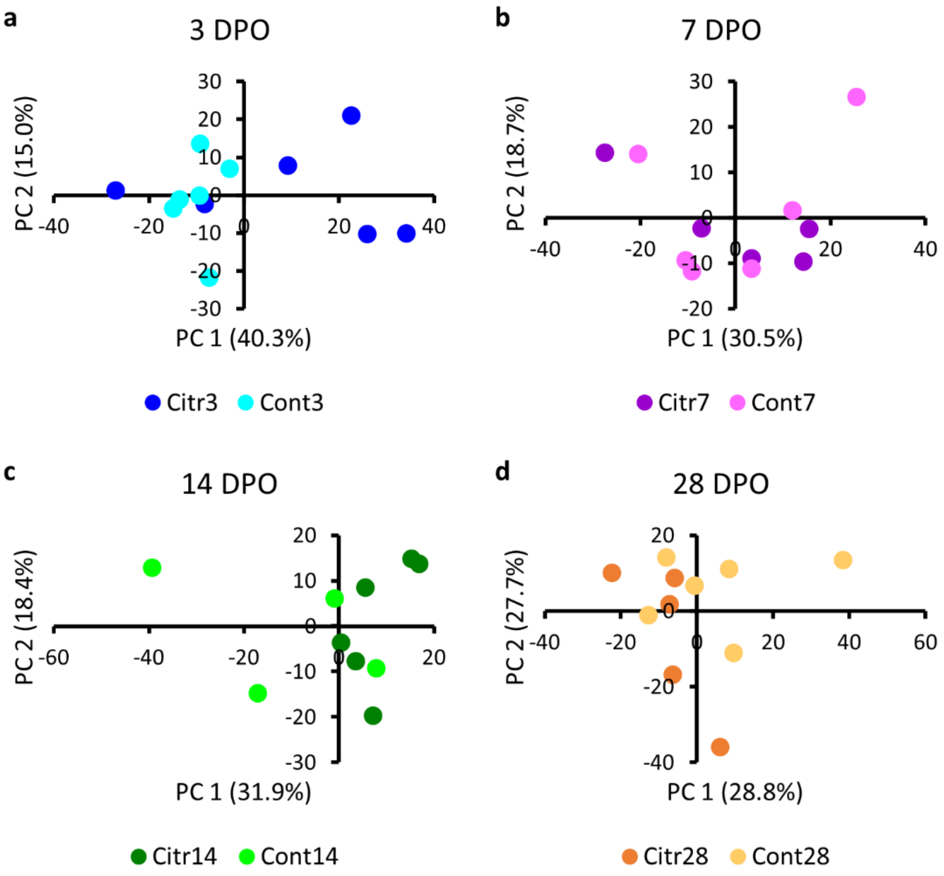

Supporting Figure S7: Principal component analyses (PCAs) of protein profiles of the citrulline supplementation (Citr) and control groups (Cont) per time point. Each dot represents one sample. The first two principal components are shown, namely PC1 and PC2, for **a** 3, **b** 7, **c** 14, and **d** 28 DPO.

Supporting Figure S8 – Volcano plots of the differentially expressed proteins

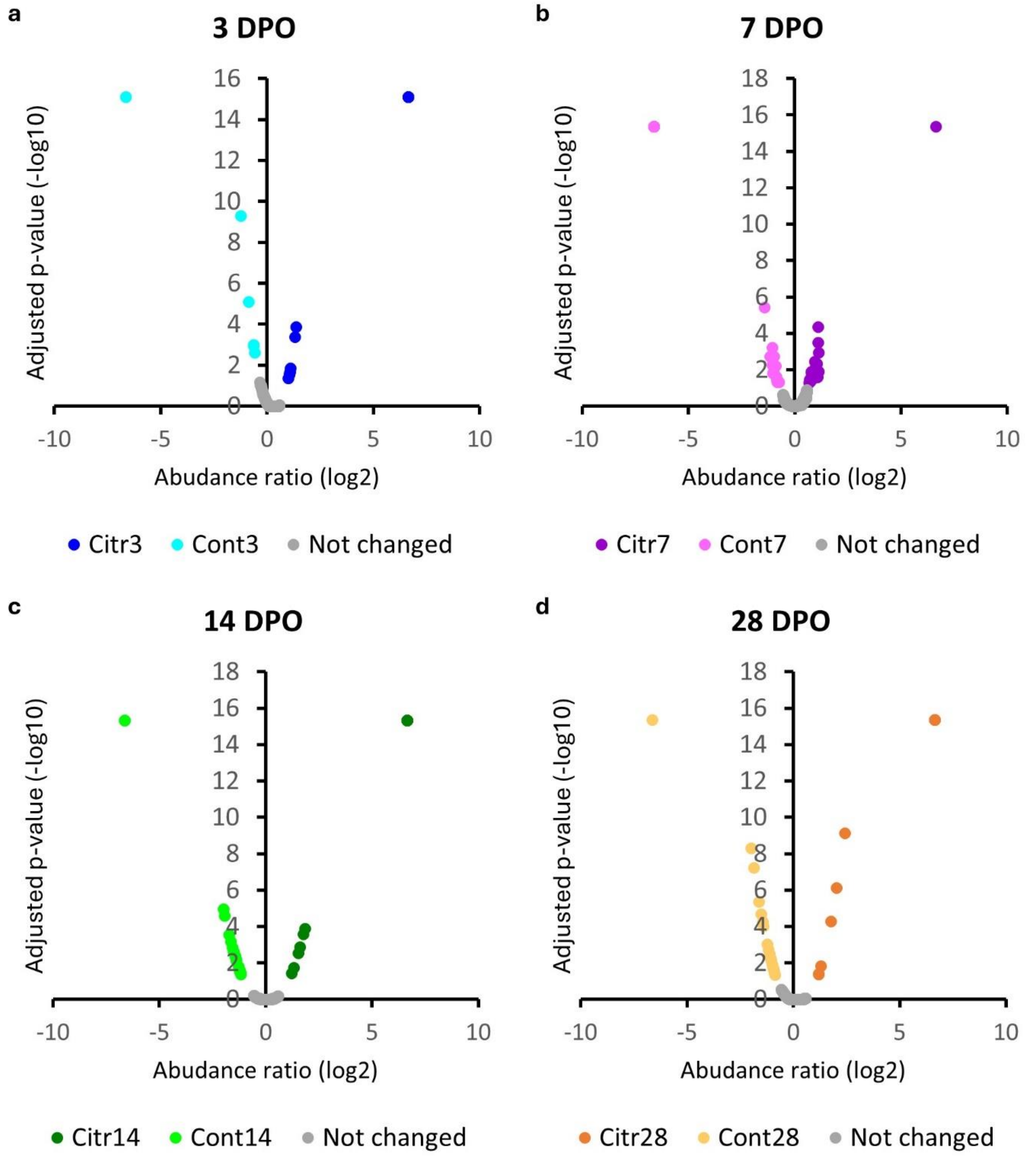

Supporting Figure S8: Volcano plots of the differently expressed proteins (3, 7, 14, and 28 DPO) for citrulline supplementation (Citr) and control (Cont) group. The differently expressed proteins (a fold change of 1.5 (log2 of ≥0.58 or ≤-0.58) and an adjusted p-value of ≤0.05) are shown for **a** 3, **b** 7, **c** 14, and **d** 28 DPO. An overview of the differently expressed proteins can be found in Supporting Table S5.

Supporting Figure S9 - Venn diagrams of the differentially expressed proteins

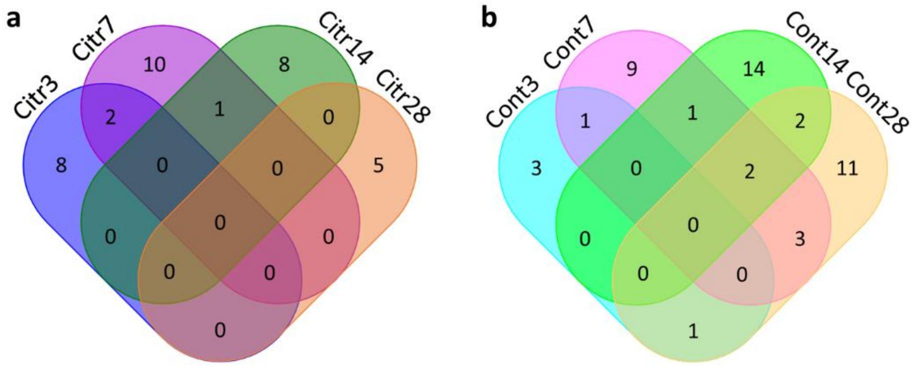

Supporting Figure S9: Venn diagrams of the differentially expressed proteins at the different time points (3, 7, 14, and 28 DPO) for citrulline supplementation (Citr) and control (Cont) group. **a** Overlap in the differentially expressed proteins in the Citr group in comparison to the respective Cont group. **b** Overlap in the differentially expressed proteins in the Cont group in comparison to the respective Citr group. The numbers depicted in the overlapping areas indicate the higher abundance of the same protein at different time points.

Supporting Figure S10 - Venn diagrams of the more active pathways

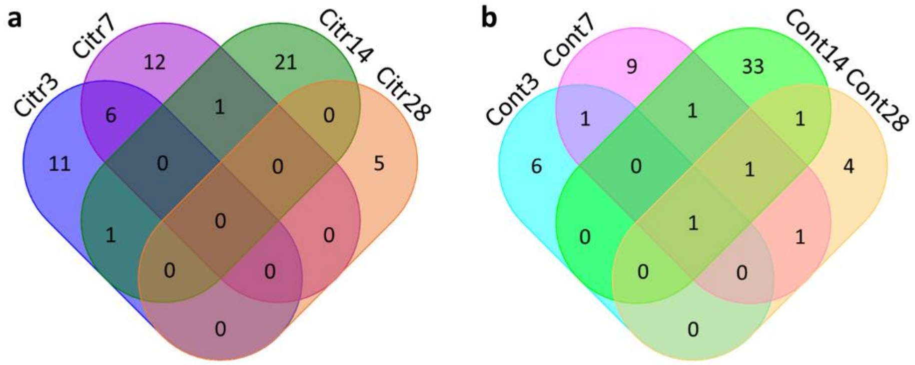

Supporting Figure S10: Venn diagrams of the more active pathways at the different time points (3, 7, 14, and 28 DPO) for citrulline supplementation (Citr) and control (Cont) group. **a** Overlap in the more active pathways in the Citr group in comparison to the respective Cont group. **b** Overlap in the more active pathways in the Cont group in comparison to the respective Citr group. The numbers depicted in the overlapping areas indicate the higher activity of the same pathway at different time points.

Supporting Table S1 - Overview of sample group references and number of samples

Supporting Table S1: Overview of sample group references and number of samples collected from citrulline supplementation rat study.

Days post-operative	Citrulline supplementation group	Control group
3	Citr3: 6 samples	Cont3: 6 samples
7	Citr7: 6 samples	Cont7: 6 samples
14	Citr14: 6 samples	Cont14: 4 samples
28	Citr28: 5 samples	Cont28: 6 samples
42	Citr42: 6 samples	Cont42: 6 samples

Supporting Table S2 - Biomechanical testing results for the citrulline supplementation and control groups

Supporting Table S2: Biomechanical testing results at the fractured and control side for the citrulline supplementation (Citr) and control (Cont) group at 42 DPO. Results are presented as mean ± STD load to failure in Newton. * indicates a significant difference with a p-value <0.05.

	Citr	Cont	p-value
**Fractured side**	160.9 ± 74.3	155.5 ± 9.3	0.730
**Control side**	267.1 ± 72.5	251.8 ± 10.5	1.000
**p-value**	0.095	0.029*	

Supporting Table S3 - Lipid assignments for bone

Supporting Table S3: Lipid assignments based on high mass resolution data and MS/MS data for bone. For each assignment, the *m/z* value obtained with the high mass resolution mass spectrometer (Solarix), the lipid assignment, the detected ion, the mass accuracy (Δ ppm error), and the sample group, in which the assigned lipid has a higher presence, are provided. The higher lipid contribution for the citrulline supplementation (Citr) group is in comparison to the control (Cont) group at one of the time points (3, 7, 14, and 28 DPO) and vice versa.

***m/z* value**	**Assignment**	**Ion**	**Mass accuracy (ppm)**	**Sample group**
400.343	CAR 16:0	$[M+H]^+$	2.25	Citr3
				Citr28
426.359	CAR 18:1	$[M+H]^+$	2.81	Cont14
				Citr28
478.330	LPC O-16:2	$[M+H]^+$	1.67	Citr3
				Citr7
				Citr28
496.341	LPC 16:0	$[M+H]^+$	2.42	Cont7
504.346	LPC O-18:3	$[M+H]^+$	2.18	Citr3
				Citr28
506.362	LPC O-18:2	$[M+H]^+$	2.96	Citr3
				Citr7
				Citr28
522.357	LPC 18:1	$[M+H]^+$	3.06	Citr3
524.372	LPC 18:0	$[M+H]^+$	1.72	Cont7
				Cont14
546.354	LPC 18:0	$[M+Na]^+$	1.83	Cont14
562.328	LPC 18:0	$[M+K]^+$	1.96	Cont14
703.577	SM 34:1;O2	$[M+H]^+$	3.13	Cont3
				Cont7
				Citr14
706.540	PC 30:0	$[M+H]^+$	2.69	Cont3
				Citr14
725.558	SM 34:1;O2	$[M+Na]^+$	1.65	Cont3
				Citr14
732.556	PC 32:1	$[M+H]^+$	3.00	Cont7
				Citr14
734.570	PC 32:0	$[M+H]^+$	0.82	Cont7
				Citr14
756.554	PC 32:0	$[M+Na]^+$	3.44	Citr14
				Cont28
758.571	PC 34:2	$[M+H]^+$	2.64	Cont7
				Cont28
760.587	PC 34:1	$[M+H]^+$	2.50	Cont3
				Cont7
				Citr14
772.527	PC 32:0	$[M+K]^+$	2.20	Citr14

Continuation Supporting Table S3 (continued): Lipid assignments for bone.

m/z value	Assignment	Ion	Mass accuracy (ppm)	Sample group
780.553	PC 34:2	$[M+Na]^+$	2.05	Cont3
				Cont28
782.571	PC 36:4	$[M+H]^+$	2.04	Cont3
				Cont7
				Citr14
				Cont28
804.554	PC 36:4	$[M+H]^+$	3.23	Cont3
808.587	PC 38:5	$[M+H]^+$	2.35	Cont3
810.603	PC 38:4	$[M+H]^+$	2.84	Cont3
				Cont7
820.528	PC 36:4	$[M+K]^+$	3.29	Cont3
832.585	PC 38:4	$[M+Na]^+$	2.76	Cont3
853.730	TG 52:5	$[M+H]^+$	2.34	Cont28
879.745	TG 54:6	$[M+H]^+$	1.59	Cont28

Supporting Table S4 - Lipid assignments for bone marrow

Supporting Table S4: Lipid assignments based on high mass resolution data and MS/MS data for bone marrow. For each assignment, the *m/z* value obtained with the high mass resolution mass spectrometer (Solarix), the lipid assignment, the detected ion, the mass accuracy (Δ ppm error), and the sample group, in which the assigned lipid has a higher presence, are provided. The higher lipid contribution for the citrulline supplementation (Citr) group is in comparison to the control (Cont) group at one of the time points (3, 7, 14, and 28 DPO) and vice versa. No identifications were included for the Citr28, as the top 30 unscaled loadings included only the isotopes of molecules and unidentified peaks.

m/z value	Assignment	Ion	Mass accuracy (ppm)	Sample group
400.343	CAR 16:0	$[M+H]^+$	2.25	Citr14
478.330	LPC O-16:2	$[M+H]^+$	1.67	Citr3
496.341	LPC 16:0	$[M+H]^+$	2.42	Citr3
				Citr14
504.346	LPC O-18:3	$[M+H]^+$	2.18	Citr3
506.362	LPC O-18:2	$[M+H]^+$	2.96	Citr3
518.323	LPC 16:0	$[M+Na]^+$	2.51	Cont7
522.357	LPC 18:1	$[M+H]^+$	3.06	Citr3
				Citr14
524.372	LPC 18:0	$[M+H]^+$	1.72	Citr14
534.297	LPC 16:0	$[M+K]^+$	2.62	Citr14
546.354	LPC 18:0	$[M+Na]^+$	1.83	Cont7
703.577	SM 34:1;O2	$[M+H]^+$	3.13	Cont7
				Citr14
706.540	PC 30:0	$[M+H]^+$	2.69	Citr3
				Citr14
720.592	PC O-32:0	$[M+H]^+$	2.78	Cont7
734.570	PC 32:0	$[M+H]^+$	0.82	Cont7
				Citr14
754.538	PC 32:1	$[M+Na]^+$	3.05	Citr7
756.554	PC 32:0	$[M+Na]^+$	3.44	Cont7
758.571	PC 34:2	$[M+H]^+$	2.64	Citr7
				Cont28
766.578	PC O-36:5	$[M+H]^+$	4.57	Cont3
				Citr7
768.592	PC O-36:4	$[M+H]^+$	2.34	Cont7
778.541	PC 36:6	$[M+H]^+$	3.72	Citr7
				Cont14
				Cont28
802.540	PC 38:8	$[M+H]^+$	2.37	Citr7
				Cont14
				Cont28
804.554	PC 36:4	$[M+Na]^+$	3.23	Cont3
				Cont14
806.572	PC 38:6	$[M+H]^+$	3.22	Cont14
				Cont28

Supporting Table S4 (continued): Lipid assignments for bone marrow.

m/z value	Assignment	Ion	Mass accuracy (ppm)	Sample group
808.587	PC 38:5	$[M+H]^+$	2.35	Cont3
810.603	PC 38:4	$[M+H]^+$	2.84	Cont7
820.528	PC 36:4	$[M+K]^+$	3.29	Cont3
				Cont14
				Cont28
828.557	PC 40:9	$[M+H]^+$	3.86	Cont3
				Citr7
				Cont14
				Cont28
830.572	PC 40:8	$[M+H]^+$	3.13	Cont3
				Cont28
832.585	PC 38:4	$[M+Na]^+$	2.76	Cont3
853.730	TG 52:5	$[M+H]^+$	2.34	Cont28
856.588	PC 42:9	$[M+H]^+$	3.39	Cont3
				Cont14
879.745	TG 54:6	$[M+H]^+$	1.59	Cont28

Supporting Table S5 - Proteins with higher abundance for the citrulline supplementation and control groups

Supporting Table S5: Proteins with higher abundance from the comparison of citrulline supplementation (Citr) group with the respective control (Cont) group per time point. Only proteins with an abundance ratio (log2) of ≥0.58 or ≤-0.58 (fold change of 1.5) and an adjusted p-value of ≤0.05 were considered differentially expressed in the corresponding sample group. Positive and negative abundance ratios were related to proteins in the Citr and Cont group, respectively. The protein name, related gene name, sample group in which the protein is differentially expressed, the abundance ratio (log2), and adjusted p-value are provided.

Supporting Table S5A Differentially expressed proteins for 3 DPO in the Citr and Cont group.

Protein name	Gene name	Sample group	Abundance ratio (log2)	Adjusted p-value
60S ribosomal protein L27	Rpl27	Citr3	1.03	0.028
Actin, alpha cardiac muscle 1	Actc1	Citr3	1.03	0.028
ADP-ribosylation factor-like protein 3	Arl3	Cont3	-6.64	$8.071e^{-16}$
Collagen alpha-1(XII) chain (Fragment)	Col12a1	Citr3	1.31	$4.258e^{-4}$
Fibromodulin	Fmod	Citr3	1.06	0.022
Hemogen	Hemgn	Citr3	6.64	$8.071e^{-16}$
Histone H3.3	H3-3b	Citr3	6.64	$8.071e^{-16}$
Keratin, type II cytoskeletal 5	Krt5	Cont3	-0.64	$9.643e^{-4}$
Leukemia inhibitory factor receptor	Lifr	Cont3	-1.23	$5.166e^{-10}$
Myosin light chain 4	Myl4	Cont3	-0.58	$2.404e^{-3}$
Nexilin	Nexn	Citr3	1.09	0.014
N(G),N(G)-dimethylarginine dimethylaminohydrolase 2	Ddah2	Citr3	0.99	0.043
Plasma kallikrein	Klkb1	Cont3	-0.63	$1.145e^{-3}$
Serine/arginine-rich splicing factor 6	Srsf6	Citr3	1.36	$1.372e^{-4}$
Slit homolog 1 protein	Slit1	Citr3	6.64	$8.071e^{-16}$

Supporting Table S5B Differentially expressed proteins for 7 DPO in the Citr and Cont group.

Protein name	Gene name	Sample group	Abundance ratio (log2)	Adjusted p-value
60S acidic ribosomal protein P1	Rplp1	Citr7	0.7	0.033
Acidic leucine-rich nuclear phosphoprotein 32 family member B	Anp32b	Cont7	-0.88	0.024
ADP-ribosylation factor-like protein 3	Arl3	Cont7	-6.64	$4.404e^{-16}$
Alpha-1B-glycoprotein	A1bg	Citr7	0.78	0.012
Band 3 anion transport protein	Slc4a1	Citr7	0.99	$6.731e^{-3}$
Calnexin	Canx	Citr7	0.93	$3.609e^{-3}$
Carbonic anhydrase 3	Ca3	Cont7	-0.91	$6.037e^{-3}$
Carboxylesterase 1D	Ces1d	Cont7	-1.03	0.015
Cathepsin G	Ctsg	Citr7	1.1	$4.266e^{-5}$
Eukaryotic translation initiation factor 3 subunit A	Eif3a	Cont7	-1.16	$1.857e^{-3}$
Galectin-5	Lgals5	Citr7	0.66	0.050
Glycogen phosphorylase, muscle form	Pygm	Citr7	1.01	0.013
Guanine deaminase	Gda	Citr7	1.08	$3.179e^{-4}$
Histone H3.3	H3-3b	Citr7	1.06	0.026
Junctophilin-2	Jph2	Citr7	1.05	$4.730e^{-3}$
Mitochondrial fission 1 protein	Fis1	Cont7	-1.05	$5.352e^{-3}$
Myosin light chain 3	Myl3	Cont7	-0.76	0.043
Myosin regulatory light chain 2, ventricular/cardiac muscle isoform	Myl2	Cont7	-1.05	$5.819e^{-4}$
NADH-ubiquinone oxidoreductase 75 kDa subunit, mitochondrial	Ndufs1	Citr7	0.73	0.043
Neutrophil gelatinase-associated lipocalin	Lcn2	Citr7	0.77	0.014
Nucleolar protein 3	Nol3	Cont7	-0.85	0.039
Proteasome subunit beta type-2	Psmb2	Cont7	-6.64	$4.404e^{-16}$
Protein phosphatase 1G	Ppm1g	Cont7	-6.64	$4.404e^{-16}$
Protein S100-B	S100b	Cont7	-0.82	0.047
Slit homolog 1 protein	Slit1	Citr7	6.64	4.404
Spondin-1	Spon1	Cont7	-0.75	0.047
Thrombospondin-4	Thbs4	Cont7	-1.44	$3.615e^{-6}$
Troponin T, slow skeletal muscle	Tnnt1	Cont7	-0.98	$1.857e^{-3}$
Vitamin K-dependent protein C	Proc	Cont7	-1.01	0.013

Supporting Table S5C Differentially expressed proteins for 14 DPO in the Citr and Cont group.

Protein name	Gene name	Sample group	Abundance ratio (log2)	Adjusted p-value
Acyl-protein thioesterase 1	Lypla1	Citr14	1.31	0.017
Calmodulin-3	Calm3	Cont14	-1.17	0.041
Cathepsin G	Ctsg	Cont14	-1.18	0.036
Collagen alpha-1(II) chain	Col2a1	Cont14	-1.93	$2.401e^{-5}$
Deoxyuridine 5'-triphosphate nucleotidohydrolase	Dut	Cont14	-1.38	$7.582e^{-3}$
Dynein light chain roadblock-type 1	Dynlrb1	Cont14	-1.29	0.015
Eukaryotic translation initiation factor 3 subunit A	Eif3a	Cont14	-1.45	$4.052e^{-3}$
Ferritin light chain 1	Ftl1	Cont14	-1.52	$2.179e^{3}$
Fructose-bisphosphate aldolase A	Aldoa	Citr14	1.21	0.036
Gamma-enolase	Eno2	Cont14	-1.22	0.028
Glucose-6-phosphate isomerase	Gpi	Citr14	1.61	$1.237e^{-3}$
Glycogen phosphorylase, muscle form	Pygm	Citr14	6.64	$4.596e^{-16}$
Keratin, type II cytoskeletal 1	Krt1	Cont14	-2.00	$1.057e^{-5}$
Lactadherin	Mfge8	Cont14	-1.57	$1.401e^{-3}$
Leukocyte cell-derived chemotaxin 1	Cnmd	Cont14	-1.72	$2.755e^{-4}$
Myosin-7	Myh7	Cont14	-1.49	$2.780e^{-3}$
Myosin light chain 3	Myl3	Cont14	-1.22	0.030
Myosin regulatory light chain 2, ventricular/cardiac muscle isoform	Myl2	Cont14	-1.44	$4.312e^{-3}$
NADH dehydrogenase [ubiquinone] iron-sulfur protein 4, mitochondrial	Ndufs4	Citr14	1.52	$2.778e^{-3}$
Nuclear autoantigenic sperm protein	Nasp	Cont14	-6.64	$4.596e^{-16}$
Phosphoglucomutase-1	Pgm1	Citr14	1.77	$2.429e^{-4}$
Programmed cell death protein 6	Pdcd6	Cont14	-1.24	0.024
Proteasomal ubiquitin receptor ADRM1	Adrm1	Citr14	6.64	$4.596e^{-16}$
Proteasome subunit beta type-2	Psmb2	Citr14	6.64	$4.596e^{-16}$
Protein RoBo-1 (Rodent bone protein)	LOC24906	Cont14	-1.64	6.362e-4
Protein S100-A8	S100a8	Cont14	-1.51	$2.324e^{-3}$
Slit homolog 1 protein	Slit1	Cont14	-6.64	$4.596e^{-16}$
Vomeronasal type-1 receptor 95	Vom1r95	Citr14	1.83	$1.245e^{-4}$

Supporting Table S5D Differentially expressed proteins for 28 DPO in the Citr and Cont group.

Protein name	Gene name	Sample group	Abundance ratio (log2)	Adjusted p-value
Alpha-crystallin B chain	Cryab	Cont28	-1.00	0.012
Annexin A6	Anxa6	Citr28	1.17	0.041
Carbonic anhydrase 3	Ca3	Cont28	-1.09	$5.002e^{-3}$
Desmin	Des	Cont28	-0.92	0.027
Elongation factor 1-alpha 2	Eef1a2	Cont28	-0.88	0.037
Ferritin light chain 1	Ftl1	Cont28	-0.96	0.017
Follistatin-related protein 1	Fstl1	Citr28	6.64	$4.222e^{-16}$
Guanine deaminase	Gda	Cont28	-0.95	0.019
Heat shock protein beta-6	Hspb6	Cont28	-1.05	$6.933e^{-3}$
Heat shock protein beta-7 (Fragment)	Hspb7	Cont28	-1.14	$2.940e^{-3}$
Heat shock protein beta-8	Hspb8	Cont28	-1.08	$5.254e^{-3}$
Keratin, type I cytoskeletal 14	Krt14	Citr28	2.41	$7.092e^{-10}$
Keratin, type II cytoskeletal 5	Krt5	Cont28	-1.12	$3.273e^{-3}$
Myosin-6	Myh6	Cont28	-2.00	$4.814e^{-9}$
Myosin-7	Myh7	Cont28	-1.62	$4.272e^{-6}$
Myosin-binding protein C, slow-type (Fragment)	Mybpc1	Cont28	-1.45	$5.367e^{-5}$
Myosin light chain 3	Myl3	Cont28	-1.51	$2.025e^{-5}$
Myosin regulatory light chain 2, ventricular/cardiac muscle isoform	Myl2	Cont28	-1.87	$5.466e^{-8}$
Myristoylated alanine-rich C-kinase substrate	Marcks	Citr28	1.18	0.039
Protein disulfide isomerase Creld2	Creld2	Citr28	1.29	0.014
Serine/arginine-rich splicing factor 2	Srsf2	Cont28	-0.86	0.043
Thrombospondin-4	Thbs4	Cont28	-1.24	$8.924e^{-4}$
Troponin I, slow skeletal muscle	Tnni1	Cont28	-1.18	$1.860e^{-3}$
Troponin T, slow skeletal muscle	Tnnt1	Cont28	-1.41	$9.272e^{-5}$

Supporting Table S6 - More active pathways for citrulline supplementation and control groups

Supporting Table S6: More active reactome pathways from the comparison of citrulline supplementation (Citr) group with the respective control (Cont) group per time point based on the differentially expressed proteins. Only pathways with a p-value of ≤0.05 were considered more activated in the corresponding sample group. The pathway name, p-value, false discovery ratio (FDR), and matched gene names are provided per time point.

Supporting Table S6A More active reactome pathways for 3 DPO in the Citr and Cont group.

Citr group				Cont group			
Pathway name	p-value	FDR	Gene names	Pathway name	p-value	FDR	Gene names
RNA Polymerase II Transcription Termination	0.002	0.086	Srsf6	RUNX1 regulates transcription of genes involved in interleukin signaling	<0.001	<0.001	Lifr
eNOS activation	0.010	0.086	Ddah2	Formation of Fibrin Clot (Clotting Cascade)	<0.001	0.002	Klkb1
Signaling by ROBO receptors	0.017	0.086	Slit1, Rpl27	Trafficking of myristoylated proteins to the cilium	0.004	0.012	Arl3
Processing of Capped Intron-Containing Pre-mRNA	0.022	0.086	Srsf6	Keratinization	0.007	0.020	Krt5
RNA Polymerase I Promoter Opening	0.030	0.086	H3-3b	Interleukin-6 family signaling	0.018	0.033	Lifr
DNA methylation	0.032	0.086	H3-3b	Activation of Matrix Metalloproteinases	0.021	0.033	Klkb1
Keratan sulfate/keratin metabolism	0.032	0.086	Fmod	Striated Muscle Contraction	0.024	0.033	Myl4
Striated Muscle Contraction	0.034	0.086	Actc1	Retrograde transport at the Trans-Golgi-Network	0.032	0.033	Arl3
Activated PKN1 stimulates transcription of AR (androgen receptor) regulated genes KLK2 and KLK3	0.034	0.086	H3-3b				
SIRT1 negatively regulates rRNA expression	0.034	0.086	H3-3b				
Assembly of the ORC complex at the origin of replication	0.035	0.086	H3-3b				
Synthesis of PA	0.036	0.086	Nexn				
PRC2 methylates histones and DNA	0.039	0.086	H3-3b				
Collagen chain trimerization	0.041	0.086	Col12a1				
Condensation of Prophase Chromosomes	0.042	0.086	H3-3b				
ERCC6 (CSB) and EHMT2 (G9a) positively regulate rRNA expression	0.042	0.086	H3-3b				
Inhibition of DNA recombination at telomere	0.045	0.086	H3-3b				
Netrin-1 signaling	0.046	0.086	Slit1				

Supporting Table S6B More active reactome pathways for 7 DPO in the Citr and Cont group.

Citr group				Cont group			
Pathway name	p-value	FDR	Gene names	Pathway name	p-value	FDR	Gene names
Glycogen breakdown (glycogenolysis)	<0.001	0.066	Pygm	Striated Muscle Contraction	<0.001	0.007	Tnnt1, Myl2, Myl3
Neutrophil degranulation	0.002	0.103	Ctsg, Pygm, A1bg, Lcn2	Reversible hydration of carbon dioxide	<0.001	0.027	Ca3
Activation, myristolyation of BID and translocation to mitochondria	0.004	0.116	Ctsg	Reactions specific to the hybrid N-glycan synthesis pathway	0.004	0.147	Myl2
Signaling by Interleukins, incl. IL-1, IL-4, IL-13, IL-27, IL-35	0.005	0.116	Crsg, Canx, Lcn2	Signaling by ERBB4	0.006	0.147	S100b
Antimicrobial peptides	0.008	0.116	Ctsg, Lcn2	Trafficking of myristoylated proteins to the cilium	0.010	0.147	Arl3
Bicarbonate transporters	0.016	0.116	Slc4a1	Interleukin-1 signaling	0.014	0.147	Psmb2, S100b
Coenzyme A biosynthesis	0.023	0.116	Gda	Signaling by NTRK1 (TRKA)	0.018	0.147	Arc
O2/CO2 exchange in erythrocytes	0.027	0.116	Slc4a1	Advanced glycosylation endproduct receptor signaling	0.023	0.147	S100b
Signaling by ROBO receptors	0.028	0.116	Rplp1, Slit1	Gamma-carboxylation, transport, and amino-terminal cleavage of proteins	0.023	0.147	Proc
Metabolism of Angiotensinogen to Angiotensins	0.030	0.116	Ctsg	Physiological factors	0.026	0.147	Ces1d
RNA Polymerase I Promoter Opening	0.037	0.116	H3-3b	Class I peroxisomal membrane protein import	0.029	0.147	Fis1
Pyroptosis	0.037	0.116	Ctsg	Common Pathway of Fibrin Clot Formation	0.036	0.147	Proc
Activation of Matrix Metalloproteinases	0.039	0.116	Ctsg	Metabolism of Angiotensinogen to Angiotensins	0.039	0.147	Ces1d
DNA methylation	0.040	0.116	H3-3b	TRAF6 mediated NF-κB activation	0.043	0.147	S100b
Signaling by NOTCH2	0.042	0.116	Ctsg				
Assembly of the ORC complex at the origin of replication	0.044	0.116	H3-3b				
Calnexin/calreticulin cycle	0.045	0.116	Canx				
PRC2 methylates histones and DNA	0.048	0.116	H3-3b				
SIRT1 negatively regulates rRNA expression	0.049	0.116	H3-3b				

Supporting Table S6C More active reactome pathways for 14 DPO in the Citr and Cont group.

Citr group				Cont group			
Pathway name	p-value	FDR	Gene names	Pathway name	p-value	FDR	Gene names
Glycogen metabolism	<0.001	<0.001	Pygm, Pgm1	Striated Muscle Contraction	<0.001	0.012	Myl2, Myl3, Myh7
Neutrophil degranulation	0.001	0.015	Gpi, Pygm, Aldoa, Pgm1	Reactions specific to the hybrid N-glycan synthesis pathway	0.002	0.112	Myl2
Glucose metabolism	0.003	0.049	Gpi, Aldoa	Regulation of cortical dendrite branching	0.008	0.112	Slit1
Galactose catabolism	0.003	0.052	Pgm1	Cam-PDE 1 activation	0.008	0.112	Calm3
eNOS activation	0.009	0.073	Lypla1	Activation, myristolyation of BID and translocation to mitochondria	0.008	0.112	Ctsg
Deubiquitination	0.023	0.073	Adrm1, Psmb2	Neutrophil degranulation	0.014	0.112	Ftl1, Ctsg, Krt1, S100a8
RAF/MAP kinase cascade	0.025	0.073	Lypla1, Psmb2	Activation of RAC1 downstream of NMDARs	0.014	0.112	Calm3
Regulation of activated PAK-2p34 by proteasome mediated degradation	0.042	0.073	Psmb2	Antimicrobial peptides	0.015	0.112	Ctsg, S100a8
Cross-presentation of soluble exogenous antigens (endosomes)	0.042	0.073	Psmb2	CREB1 phosphorylation through the activation of CaMKII/CaMKK/CaMKIV cascade	0.016	0.112	Calm3
Regulation of ornithine decarboxylase (ODC)	0.043	0.073	Psmb2	Glucose metabolism	0.016	0.112	Eno2
GSK3B and BTRC:CUL1-mediated-degradation of NFE2L2	0.044	0.073	Psmb2	Calcineurin activates NFAT	0.018	0.112	Calm3
p53-Independent DNA Damage Response	0.044	0.073	Psmb2	Activation of Ca-permeable Kainate Receptor	0.020	0.112	Calm3
Ubiquitin-dependent degradation of Cyclin D	0.044	0.073	Psmb2	CaMK IV-mediated phosphorylation of CREB	0.020	0.112	Calm3
Autodegradation of the E3 ubiquitin ligase COP1	0.044	0.073	Psmb2	Tetrahydrobiopterin (BH4) synthesis, recycling, salvage and regulation	0.020	0.112	Calm3
FBXL7 down-regulates AURKA during mitotic entry and in early mitosis	0.046	0.073	Psmb2	Regulation of commissural axon pathfinding by SLIT and ROBO	0.020	0.112	Slit1
SCF-beta-TrCP mediated degradation of Emi1	0.046	0.073	Psmb2	CLEC7A (Dectin-1) induces NFAT activation	0.022	0.112	Calm3
Degradation of AXIN	0.046	0.073	Psmb2	eNOS activation	0.022	0.112	Calm3
Negative regulation of NOTCH4 signaling	0.046	0.073	Psmb2	Sodium/Calcium exchangers	0.022	0.112	Calm3
Regulation of RUNX3 expression and activity	0.046	0.073	Psmb2	CREB1 phosphorylation through the activation of Adenylate Cyclase	0.023	0.112	Calm3
AUF1 (hnRNP D0) binds and destabilizes mRNA	0.047	0.073	Psmb2	Reduction of cytosolic Ca++ levels	0.023	0.112	Calm3

Supporting Table S6C (continued) More active reactome pathways for 14 DPO in the Citr and Cont group.

**Citr group**				**Cont group**			
Pathway name	p-value	FDR	Gene names	Pathway name	p-value	FDR	Gene names
Degradation of DVL	0.048	0.073	Psmb2	NOTCH2 intracellular domain regulates transcription	0.023	0.112	Ctsg
Complex I biogenesis	0.048	0.073	Ndufs4	Regulation of Insulin-like Growth Factor (IGF) transport and uptake by Insulin-like Growth Factor Binding Proteins (IGFBPs)	0.025	0.112	Ctsg, Mfge8
NIK-->noncanonical NF-κB signaling	0.050	0.073	Psmb2	Degradation of the extracellular matrix	0.031	0.112	Col2a1, Ctsg
				Protein methylation	0.033	0.112	Calm3
				PKA activation	0.035	0.112	Calm3
				Metabolism of Angiotensinogen to Angiotensins	0.035	0.112	Ctsg
				Scavenging by Class A Receptors	0.037	0.112	Ftl1
				VEGFR2 mediated cell proliferation	0.039	0.112	Calm3
				Negative regulation of NMDA receptor-mediated neuronal transmission	0.041	0.112	Calm3
				Ras activation upon Ca2+ influx through NMDA receptor	0.041	0.112	Calm3
				Unblocking of NMDA receptors, glutamate binding and activation	0.041	0.112	Calm3
				RHO GTPases activate PAKs	0.041	0.112	Calm3
				Regulation of TLR by endogenous ligand	0.041	0.112	S100a8
				DARPP-32 events	0.046	0.112	Calm3
				RHO GTPases Activate NADPH Oxidases	0.046	0.112	S100a8
				Long-term potentiation	0.048	0.112	Calm3
				Glycogen metabolism	0.048	0.112	Calm3

Supporting Table S6D More active reactome pathways for 28 DPO in the Citr and Cont group.

Citr group				Cont group			
Pathway name	p-value	FDR	Gene names	Pathway name	p-value	FDR	Gene names
Keratinization	0.005	0.023	Krt14	Striated Muscle Contraction	<0.001	<0.001	Des, Mybpc1, Myl2, Myl3, Myh6, Myh7, Tnni1, Tnnt1
Acetylcholine regulates insulin secretion	0.005	0.023	Marcks	Reversible hydration of carbon dioxide	<0.001	0.006	Ca3
Type I hemidesmosome assembly	0.006	0.023	Krt14	Reactions specific to the hybrid N-glycan synthesis pathway	0.002	0.024	Myl2
Signaling by BMP	0.014	0.043	Fstl1	Coenzyme A biosynthesis	0.016	0.142	Gda
Smooth Muscle Contraction	0.023	0.055	Anxa6	Type I hemidesmosome assembly	0.022	0.151	Krt5
				Formation of the cornified envelope	0.027	0.182	Krt5
				Scavenging by Class A Receptors	0.037	0.182	Ftl1
				Purine catabolism	0.039	0.182	Gda

References

(1) Diwan, A. D.; Wang, M. X.; Jang, D.; Zhu, W.; Murrell, G. A. Nitric oxide modulates fracture healing. *J Bone Miner Res* **2000**, *15* (2), 342-351. DOI: 10.1359/jbmr.2000.15.2.342 From NLM Medline.

(2) Meesters, D. M.; Wijnands, K. A. P.; Brink, P. R. G.; Poeze, M. Malnutrition and Fracture Healing: Are Specific Deficiencies in Amino Acids Important in Nonunion Development? *Nutrients* **2018**, *10* (11). DOI: 10.3390/nu10111597 From NLM Medline.

(3) Baldik, Y.; Talu, U.; Altinel, L.; Bilge, H.; Demiryont, M.; Aykac-Toker, G. Bone healing regulated by nitric oxide: an experimental study in rats. *Clin Orthop Relat Res* **2002**, (404), 343-352. DOI: 10.1097/00003086-200211000-00051 From NLM Medline.

(4) Corbett, S. A.; Hukkanen, M.; Batten, J.; McCarthy, I. D.; Polak, J. M.; Hughes, S. P. Nitric oxide in fracture repair. Differential localisation, expression and activity of nitric oxide synthases. *J Bone Joint Surg Br* **1999**, *81* (3), 531-537. DOI: 10.1302/0301-620x.81b3.8852 From NLM Medline.

(5) Oryan, A.; Monazzah, S.; Bigham-Sadegh, A. Bone injury and fracture healing biology. *Biomed Environ Sci* **2015**, *28* (1), 57-71. DOI: 10.3967/bes2015.006 From NLM Medline.

(6) Zhu, W.; Murrell, G. A.; Lin, J.; Gardiner, E. M.; Diwan, A. D. Localization of nitric oxide synthases during fracture healing. *J Bone Miner Res* **2002**, *17* (8), 1470-1477. DOI: 10.1359/jbmr.2002.17.8.1470 From NLM Medline.

(7) Zhu, W.; Diwan, A. D.; Lin, J. H.; Murrell, G. A. Nitric oxide synthase isoforms during fracture healing. *J Bone Miner Res* **2001**, *16* (3), 535-540. DOI: 10.1359/jbmr.2001.16.3.535 From NLM Medline.

(8) Meesters, D. M.; Hannemann, P. F.; van Eijk, H. M.; Schriebl, V. T.; Brink, P. R.; Poeze, M.; Wijnands, K. A. Enhancement of fracture healing after citrulline supplementation in mice. *Eur Cell Mater* **2020**, *39*, 183-192. DOI: 10.22203/eCM.v039a12 From NLM Medline.

(9) Jiang, L.; Sun, J.; Huang, D. Role of Slit/Robo Signaling pathway in Bone Metabolism. *Int J Biol Sci* **2022**, *18* (3), 1303-1312. DOI: 10.7150/ijbs.66931 From NLM Medline.

(10) Tong, M.; Jun, T.; Nie, Y.; Hao, J.; Fan, D. The Role of the Slit/Robo Signaling Pathway. *J Cancer* **2019**, *10* (12), 2694-2705. DOI: 10.7150/jca.31877 From NLM PubMed-not-MEDLINE.

(11) Lim, H. K.; Choi, Y. A.; Park, W.; Lee, T.; Ryu, S. H.; Kim, S. Y.; Kim, J. R.; Kim, J. H.; Baek, S. H. Phosphatidic acid regulates systemic inflammatory responses by modulating the Akt-mammalian target of rapamycin-p70 S6 kinase 1 pathway. *J Biol Chem* **2003**, *278* (46), 45117-45127. DOI: 10.1074/jbc.M303789200 From NLM Medline.

(12) Allori, A. C.; Sailon, A. M.; Warren, S. M. Biological basis of bone formation, remodeling, and repair-part II: extracellular matrix. *Tissue Eng Part B Rev* **2008**, *14* (3), 275-283. DOI: 10.1089/ten.teb.2008.0083 From NLM Medline.

(13) Lin, X.; Patil, S.; Gao, Y. G.; Qian, A. The Bone Extracellular Matrix in Bone Formation and Regeneration. *Front Pharmacol* **2020**, *11*, 757. DOI: 10.3389/fphar.2020.00757 From NLM PubMed-not-MEDLINE.

(14) Dimitriou, R.; Tsiridis, E.; Giannoudis, P. V. Current concepts of molecular aspects of bone healing. *Injury* **2005**, *36* (12), 1392-1404. DOI: 10.1016/j.injury.2005.07.019 From NLM Medline.

(15) Einhorn, T. A.; Gerstenfeld, L. C. Fracture healing: mechanisms and interventions. *Nat Rev Rheumatol* **2015**, *11* (1), 45-54. DOI: 10.1038/nrrheum.2014.164 From NLM Medline.

(16) Loi, F.; Cordova, L. A.; Pajarinen, J.; Lin, T. H.; Yao, Z.; Goodman, S. B. Inflammation, fracture and bone repair. *Bone* **2016**, *86*, 119-130. DOI: 10.1016/j.bone.2016.02.020 From NLM Medline.

(17) Marsell, R.; Einhorn, T. A. The biology of fracture healing. *Injury* **2011**, *42* (6), 551-555. DOI: 10.1016/j.injury.2011.03.031 From NLM Medline.

(18) Tsiridis, E.; Upadhyay, N.; Giannoudis, P. Molecular aspects of fracture healing: which are the important molecules? *Injury* **2007**, *38 Suppl 1*, S11-25. DOI: 10.1016/j.injury.2007.02.006 From NLM Medline.

(19) Shiu, H. T.; Leung, P. C.; Ko, C. H. The roles of cellular and molecular components of a hematoma at early stage of bone healing. *J Tissue Eng Regen Med* **2018**, *12* (4), e1911-e1925. DOI: 10.1002/term.2622 From NLM Medline.

(20) Paiva, K. B.; Granjeiro, J. M. Bone tissue remodeling and development: focus on matrix metalloproteinase functions. *Arch Biochem Biophys* **2014**, *561*, 74-87. DOI: 10.1016/j.abb.2014.07.034 From NLM Medline.
(21) Chandel, N. S. Carbohydrate Metabolism. *Cold Spring Harb Perspect Biol* **2021**, *13* (1). DOI: 10.1101/cshperspect.a040568 From NLM Medline.
(22) Bastian, O. W.; Kuijer, A.; Koenderman, L.; Stellato, R. K.; van Solinge, W. W.; Leenen, L. P.; Blokhuis, T. J. Impaired bone healing in multitrauma patients is associated with altered leukocyte kinetics after major trauma. *J Inflamm Res* **2016**, *9*, 69-78. DOI: 10.2147/JIR.S101064 From NLM PubMed-not-MEDLINE.
(23) Kovtun, A.; Messerer, D. A. C.; Scharffetter-Kochanek, K.; Huber-Lang, M.; Ignatius, A. Neutrophils in Tissue Trauma of the Skin, Bone, and Lung: Two Sides of the Same Coin. *J Immunol Res* **2018**, *2018*, 8173983. DOI: 10.1155/2018/8173983 From NLM Medline.
(24) Mangoni, M. L.; McDermott, A. M.; Zasloff, M. Antimicrobial peptides and wound healing: biological and therapeutic considerations. *Exp Dermatol* **2016**, *25* (3), 167-173. DOI: 10.1111/exd.12929 From NLM Medline.
(25) Canalis, E. Notch in skeletal physiology and disease. *Osteoporos Int* **2018**, *29* (12), 2611-2621. DOI: 10.1007/s00198-018-4694-3 From NLM Medline.
(26) Ballhause, T. M.; Jiang, S.; Baranowsky, A.; Brandt, S.; Mertens, P. R.; Frosch, K. H.; Yorgan, T.; Keller, J. Relevance of Notch Signaling for Bone Metabolism and Regeneration. *Int J Mol Sci* **2021**, *22* (3). DOI: 10.3390/ijms22031325 From NLM Medline.
(27) Zieba, J. T.; Chen, Y. T.; Lee, B. H.; Bae, Y. Notch Signaling in Skeletal Development, Homeostasis and Pathogenesis. *Biomolecules* **2020**, *10* (2). DOI: 10.3390/biom10020332 From NLM Medline.
(28) Cordat, E.; Casey, J. R. Bicarbonate transport in cell physiology and disease. *Biochem J* **2009**, *417* (2), 423-439. DOI: 10.1042/BJ20081634 From NLM Medline.
(29) Mo, C.; Ke, J.; Zhao, D.; Zhang, B. Role of the renin-angiotensin-aldosterone system in bone metabolism. *J Bone Miner Metab* **2020**, *38* (6), 772-779. DOI: 10.1007/s00774-020-01132-y From NLM Medline.
(30) Garcia, P.; Schwenzer, S.; Slotta, J. E.; Scheuer, C.; Tami, A. E.; Holstein, J. H.; Histing, T.; Burkhardt, M.; Pohlemann, T.; Menger, M. D. Inhibition of angiotensin-converting enzyme stimulates fracture healing and periosteal callus formation - role of a local renin-angiotensin system. *Br J Pharmacol* **2010**, *159* (8), 1672-1680. DOI: 10.1111/j.1476-5381.2010.00651.x From NLM Medline.
(31) Chim, S. M.; Tickner, J.; Chow, S. T.; Kuek, V.; Guo, B.; Zhang, G.; Rosen, V.; Erber, W.; Xu, J. Angiogenic factors in bone local environment. *Cytokine Growth Factor Rev* **2013**, *24* (3), 297-310. DOI: 10.1016/j.cytogfr.2013.03.008 From NLM Medline.
(32) Su, Y. W.; Zhou, X. F.; Foster, B. K.; Grills, B. L.; Xu, J.; Xian, C. J. Roles of neurotrophins in skeletal tissue formation and healing. *J Cell Physiol* **2018**, *233* (3), 2133-2145. DOI: 10.1002/jcp.25936 From NLM Medline.
(33) Plotkin, L. I.; Essex, A. L.; Davis, H. M. RAGE Signaling in Skeletal Biology. *Curr Osteoporos Rep* **2019**, *17* (1), 16-25. DOI: 10.1007/s11914-019-00499-w From NLM Medline.
(34) Abu-Amer, Y. NF-kappaB signaling and bone resorption. *Osteoporos Int* **2013**, *24* (9), 2377-2386. DOI: 10.1007/s00198-013-2313-x From NLM Medline.
(35) Thouverey, C.; Caverzasio, J. Focus on the p38 MAPK signaling pathway in bone development and maintenance. *Bonekey Rep* **2015**, *4*, 711. DOI: 10.1038/bonekey.2015.80 From NLM PubMed-not-MEDLINE.
(36) Chae, H. J.; Park, R. K.; Chung, H. T.; Kang, J. S.; Kim, M. S.; Choi, D. Y.; Bang, B. G.; Kim, H. R. Nitric oxide is a regulator of bone remodelling. *J Pharm Pharmacol* **1997**, *49* (9), 897-902. DOI: 10.1111/j.2042-7158.1997.tb06132.x From NLM Medline.
(37) Rajfer, R. A.; Kilic, A.; Neviaser, A. S.; Schulte, L. M.; Hlaing, S. M.; Landeros, J.; Ferrini, M. G.; Ebramzadeh, E.; Park, S. H. Enhancement of fracture healing in the rat, modulated by compounds that stimulate inducible nitric oxide synthase: Acceleration of fracture healing via inducible nitric oxide synthase. *Bone Joint Res* **2017**, *6* (2), 90-97. DOI: 10.1302/2046-3758.62.BJR-2016-0164.R2 From NLM PubMed-not-MEDLINE.

(38) Tejero, J.; Stuehr, D. Tetrahydrobiopterin in nitric oxide synthase. *IUBMB Life* **2013**, *65* (4), 358-365. DOI: 10.1002/iub.1136 From NLM Medline.
(39) Hou, C. H.; Lin, J.; Huang, S. C.; Hou, S. M.; Tang, C. H. Ultrasound stimulates NF-kappaB activation and iNOS expression via the Ras/Raf/MEK/ERK signaling pathway in cultured preosteoblasts. *J Cell Physiol* **2009**, *220* (1), 196-203. DOI: 10.1002/jcp.21751 From NLM Medline.
(40) Kubo, Y.; Wruck, C. J.; Fragoulis, A.; Drescher, W.; Pape, H. C.; Lichte, P.; Fischer, H.; Tohidnezhad, M.; Hildebrand, F.; Pufe, T.; et al. Role of Nrf2 in Fracture Healing: Clinical Aspects of Oxidative Stress. *Calcif Tissue Int* **2019**, *105* (4), 341-352. DOI: 10.1007/s00223-019-00576-3 From NLM Medline.
(41) Chen, Y.; Alman, B. A. Wnt pathway, an essential role in bone regeneration. *J Cell Biochem* **2009**, *106* (3), 353-362. DOI: 10.1002/jcb.22020 From NLM Medline.
(42) Houschyar, K. S.; Tapking, C.; Borrelli, M. R.; Popp, D.; Duscher, D.; Maan, Z. N.; Chelliah, M. P.; Li, J.; Harati, K.; Wallner, C.; et al. Wnt Pathway in Bone Repair and Regeneration - What Do We Know So Far. *Front Cell Dev Biol* **2018**, *6*, 170. DOI: 10.3389/fcell.2018.00170 From NLM PubMed-not-MEDLINE.
(43) Noel, L. S.; Champion, B. R.; Holley, C. L.; Simmons, C. J.; Morris, D. C.; Payne, J. A.; Lean, J. M.; Chambers, T. J.; Zaman, G.; Lanyon, L. E.; et al. RoBo-1, a novel member of the urokinase plasminogen activator receptor/CD59/Ly-6/snake toxin family selectively expressed in rat bone and growth plate cartilage. *J Biol Chem* **1998**, *273* (7), 3878-3883. DOI: 10.1074/jbc.273.7.3878 From NLM Medline.
(44) Kajiya, H. Calcium Signaling in Osteoclast Differentiation and Bone Resorption. In *Calcium Signaling*, Islam, M. S. Ed.; Springer Netherlands, 2012; pp 917-932.
(45) Zayzafoon, M. Calcium/calmodulin signaling controls osteoblast growth and differentiation. *J Cell Biochem* **2006**, *97* (1), 56-70. DOI: 10.1002/jcb.20675 From NLM Medline.
(46) Lee, B. S. Myosins in Osteoclast Formation and Function. *Biomolecules* **2018**, *8* (4). DOI: 10.3390/biom8040157 From NLM Medline.
(47) Strzelecka-Kiliszek, A.; Mebarek, S.; Roszkowska, M.; Buchet, R.; Magne, D.; Pikula, S. Functions of Rho family of small GTPases and Rho-associated coiled-coil kinases in bone cells during differentiation and mineralization. *Biochim Biophys Acta Gen Subj* **2017**, *1861* (5 Pt A), 1009-1023. DOI: 10.1016/j.bbagen.2017.02.005 From NLM Medline.
(48) Schroder, K. NADPH oxidases in bone homeostasis and osteoporosis. *Free Radic Biol Med* **2019**, *132*, 67-72. DOI: 10.1016/j.freeradbiomed.2018.08.036 From NLM Medline.
(49) Ducy, P. Bone Regulation of Insulin Secretion and Glucose Homeostasis. *Endocrinology* **2020**, *161* (10). DOI: 10.1210/endocr/bqaa149 From NLM Medline.
(50) Fulzele, K.; Clemens, T. L. Novel functions for insulin in bone. *Bone* **2012**, *50* (2), 452-456. DOI: 10.1016/j.bone.2011.06.018 From NLM Medline.
(51) Agrawal, A.; Jorgensen, N. R. Extracellular purines and bone homeostasis. *Biochem Pharmacol* **2021**, *187*, 114425. DOI: 10.1016/j.bcp.2021.114425 From NLM Medline.
